# Chemical Constituents of *Anacardium occidentale* as Inhibitors of *Trypanosoma cruzi* Sirtuins

**DOI:** 10.3390/molecules24071299

**Published:** 2019-04-03

**Authors:** Tanira Matutino Bastos, Helena Mannochio Russo, Nilmar Silvio Moretti, Sergio Schenkman, Laurence Marcourt, Mahabir Prashad Gupta, Jean-Luc Wolfender, Emerson Ferreira Queiroz, Milena Botelho Pereira Soares

**Affiliations:** 1 Instituto Gonçalo Moniz, FIOCRUZ, Salvador, BA 40296-710, Brazil; tancomb@hotmail.com; 2 School of Pharmaceutical Sciences, EPGL, University of Geneva, University of Lausanne, CMU, 1, Rue Michel Servet, 1211 Geneva, Switzerland; helenamrusso@gmail.com (H.M.R.); laurence.marcourt@unige.ch (L.M.); jean-luc.wolfender@unige.ch (J.-L.W.); emerson.ferreira@unige.ch (E.F.Q.); 3 Departmento de Microbiologia, Imunologia e Parasitologia, UNIFESP, São Paulo, SP 04039-032, Brazil; nilmar.moretti@unifesp.br (N.S.M.); sergioschenkman@gmail.com (S.S.); 4 Center for Pharmacognostic Research on Panamanian Flora (CIFLORPAN), College of Pharmacy, University of Panama, Panama 0824-00172, Panama; mahabirgupta@gmail.com

**Keywords:** *Trypanosoma cruzi*, sirtuins, *Anacardium occidentale*, drug discovery

## Abstract

Benznidazole and nifurtimox, the only drugs available for the treatment of Chagas disease, have limited efficacy and have been associated with severe adverse side effects. Thus, there is an urgent need to find new biotargets for the identification of novel bioactive compounds against the parasite and with low toxicity. Silent information regulator 2 (Sir2) enzymes, or sirtuins, have emerged as attractive targets for the development of novel antitrypanosomatid agents. In the present work, we evaluated the inhibitory effect of natural compounds isolated from cashew nut (*Anacardium occidentale*, L. Anacardiaceae) against the target enzymes TcSir2rp1 and TcSir2rp3 as well as the parasite. Two derivates of cardol (**1**, **2**), cardanol (**3**, **4**), and anacardic acid (**5**, **6**) were investigated. The two anacardic acids (**5**, **6**) inhibited both TcSir2rp1 and TcSir2rp3, while the cardol compound (**2**) inhibited only TcSir2rp1. The most potent sirtuin inhibitor active against the parasite was the cardol compound (**2**), with an EC_50_ value of 12.25 µM, similar to that of benznidazole. Additionally, compounds (**1**, **4**), which were inactive against the sirtuin targets, presented anti-*T. cruzi* effects. In conclusion, our results showed the potential of *Anacardium occidentale* compounds for the development of potential sirtuin inhibitors and anti-*Trypanosoma cruzi* agents.

## 1. Introduction

Chagas disease is a neglected parasitic disease caused by infection with the protozoan *Trypanosoma cruzi* (Trypanosomatidae). About 6–7 million people live with Chagas disease worldwide, mainly in Latin America, where the disease is endemic [1]. Due to human migration, the disease has also spread to different non-endemic regions such as North America, Europe, Japan, and Australia [2]. Since the end of the 1960s and the beginning of the 1970s, two drugs, nifurtimox and benznidazole, have been used for the treatment of Chagas disease [3]. The treatment is mostly effective during the acute phase, with low efficacy in curing chronic adult patients, in addition to being associated with severe toxicity [4]. Since none of the currently available treatments are ideal, there is an urgent need to discover new drugs. Different approaches have been used for drug discovery against trypanosomatids including target-based approaches, which involves screening for inhibitors against a purified protein, compound repositioning, and phenotypic screening approaches [5].

Silent information regulator 2 (Sir2), or sirtuins, are a family of NAD^+^-dependent enzymes involved in the deacetylation of lysine residues of proteins. Sirtuins are evolutionarily conserved and present in all kingdoms of life from bacteria to higher eukaryotes. By promoting protein deacetylation, distinct sirtuin homologues regulate some key biological activities in many organisms [6,7]. In trypanosomatids, sirtuins have unique characteristics and have been shown to regulate DNA repair, telomeric gene silencing, parasite growth and differentiation, and parasite morphology remodeling [8,9,10,11,12,13,14]. Therefore, sirtuins have been proposed as potential anti-parasitic targets for drug development [15].

*T. cruzi* presents two genes coding for sirtuins, one coding for TcSir2rp1 and the other for TcSir2rp3, which are located in the cytoplasm and mitochondria, respectively [8,9]. Sirtuin inhibitors affect *T. cruzi* epimastigote growth and viability, control of parasite infection in vitro and in vivo, induce activation of apoptosis and autophagy, and when overexpressed, they protect the parasite against sirtuin inhibitors [8,9,13,16].

Molecular docking composed of a small library of phytochemicals has previously identified a restricted number of scaffolds that potentially interact with the modeled TcSir2rp1 and TcSir2rp3 enzymes including an anarcadic acid derivative obtained from the cashew nut shell liquid (CNSL), which exhibited the best overall docking results in both parasitic sirtuins [17].

Cashew (*Anacardium occidentale*) is a very well-known species from the Anarcardiaceae family that is found in tropical countries around the world such as Brazil, Nigeria, India, Indonesia, Central America and Panama, Mozambique, and Vietnam [18]. Various biological properties of CNSL have been described, mainly due to its phenolic components including antimicrobial properties, anti-inflammatory, antitumor (through suppressing angiogenesis and also histone acetyltransferase activity), molluscicidal activity, antioxidant, and insecticidal against *Aedes aegypti*; it also acts as mushroom tyrosinase inhibitors, showing, therefore, great therapeutic potential [19,20,21,22,23,24,25]. Therefore, we explored the effect of CNSL on the inhibition of *T. cruzi* sirtuins as possible antiparasitic compounds. Here, we evaluated six different compounds derived from anacardic acid, cardol, and cardanol classes isolated from *A. occidentale*, and determined their in vitro inhibitory activity against TcSir2rp1 and TcSir2rp3 as well as their antiparasitic activity.

## 2. Results

### 2.1. The Cashew Nut Isolated Compounds

The cashew nut powder was extracted successfully first with hexane to eliminate the fatty acids, followed by dichloromethane and methanol. All extracts were analyzed by HPLC-PDA to localize the expected cardol, cardanol, and anacardic acid derivatives previously described from the cashew nut [26]. The dichloromethane extract was selected for the next step of purification because of its high content of the targeted compounds.

**Figure 1:**
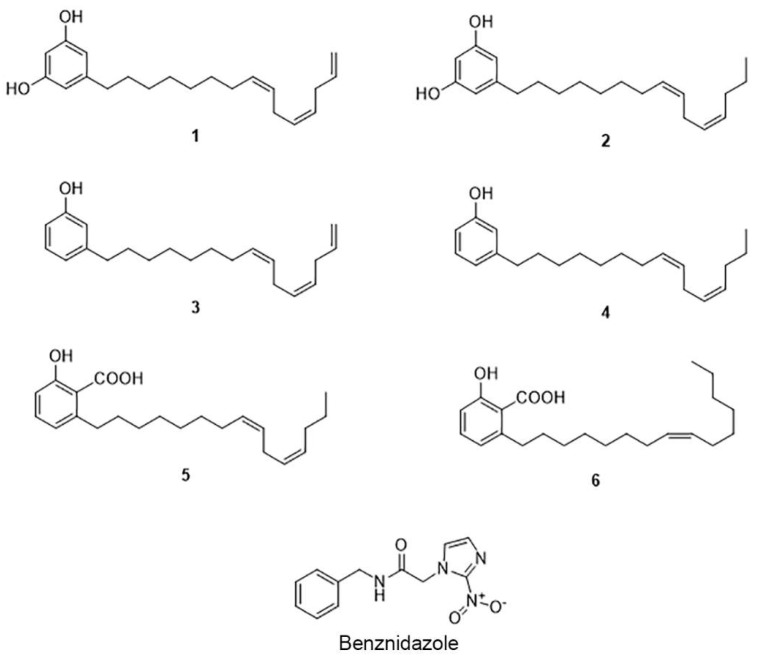
Structure of the isolated compounds and benznidazole.

In order to isolate these phenolic compounds, the cashew nut dichloromethane extract was purified by MPLC-UV. The analytical HPLC conditions were first optimized and transferred to the MPLC with a gradient transfer method [27] (Figure S1, supporting information). Using this approach, it was possible to obtain and identify three compounds in one step (**1**, **2**, and **6**) and three other compounds (**3**–**5**) with further semi preparative HPLC-UV purification (Figure 1). This approach provides a rational way to predict the retention time and resolution at the analytical scale prior to the semi-preparative separation in MPLC-UV, in comparison to previously chromatographic methods used to purify the same extract [28]. This contemporary methodology accelerates the isolation of the compounds by reducing the number of chromatographic steps and affords natural compounds with higher purity.

The NMR analysis of the isolated compounds (supporting information) was compared to the literature and confirmed the structure of the compounds as cardol triene [5-(8*Z*,11*Z*)-8,11,14-Pentadecatrien-1-yl-1,3-benzenediol] (**1**) [29], cardol diene [5-(8*Z*,11*Z*)-8,11-Pentadecadien-1-yl-1,3-benzenediol] (**2**) [29,30], cardanol triene [3-(8*Z*,11*Z*)-8,11,14-Pentadecatrien-1-ylphenol] (**3**) [30], cardanol monoene [3-(8Z)-8-Pentadecen-1-ylphenol] (**4**) [30], anacardic acid diene [2-Hydroxy-6-(8Z,11Z)-8,11-pentadecadien-1-ylbenzoic acid] (**5**) [31], and anacardic acid monoene [2-Hydroxy-6-(8Z)-8-pentadecenylbenzoic acid] (**6**) [31] (Figure 1).

### 2.2. Trypanosoma cruzi Recombinant Sirtuins In Vitro Deacetylase Activity

**Figure 2:**
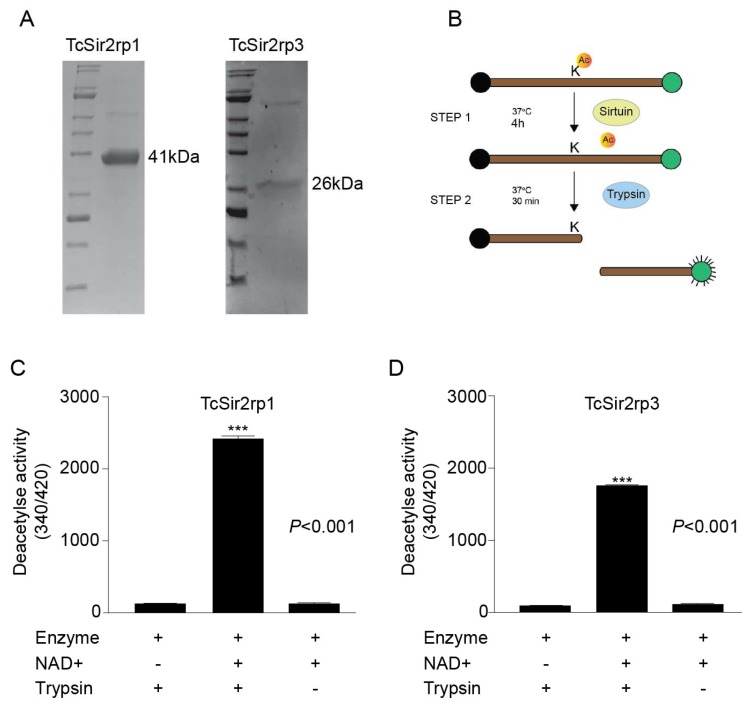
*Trypanosoma cruzi* recombinant proteins Sir2rp1 and Sir2rp3 in vitro deacetylase activity. (**A**) SDS-PAGE stained with Coomassie brilliant blue of purified TcSir2rp1 and TcSir2rp3 recombinant proteins. (**B**) Schematic representation of the deacetylation assay used to test the TcSir2rp activity. The NAD^+^-deacetylase activity of TcSir2rp1 (**C**) and TcSir2rp3 (**D**) recombinant proteins. Bars represent the mean ± standard error of the mean (SEM) of three experiments. *** *p* < 0.001.

The biological activity of the isolated compounds was evaluated against two molecular targets of *T. cruzi*: the sirtuins TcSir2rp1 and TcSir2rp3. First, we produced recombinant TcSir2rp1 and TcSir2rp3 by amplifying its genes from the Y strain *T. cruzi* genome and then cloning it into the pET-28a vector. *E. coli* transformation with the resulting plasmids produced soluble proteins. Recombinant TcSir2rp1 and TcSir3rp3 were separated from other bacterial proteins by purification using affinity chromatography and the purified proteins were run on SDS-PAGE gel stained with Coomassie blue, resulting in the expression of recombinant proteins with an estimated molecular size of 41 kDa for TcSir2rp1 and 26 kDa for TcSir2rp3 (Figure 2A).

To confirm the presence of sirtuin activity (class III deacetylases), deacetylase activity assays were performed with both recombinant enzymes (Figure 2B). As a control, we performed the reaction with and without NAD^+^, the enzyme co-factor required for deacetylation. It was expected that deacetylation of the substrate allowed cleavage of the peptide by trypsin, resulting in the separation of the quencher (EDDnp) from the fluorophore (ABZ) that became fluorescent. Indeed, in the presence of NAD^+^, we observed an increased fluorescence, indicating the deacetylation of the substrate followed by trypsin cleavage. In contrast, in the absence of NAD^+^, there was no increase in fluorescence, confirming TcSir2rp1 and TcSir2rp3 as NAD^+^-dependent deacetylases (Figure 2C,D) and validating the enzymatic assay.

Previous in silico analysis predicted that a derivate of anarcadic acid potentially interacts with modeled TcSir2rp1 and TcSir2rp3, suggesting the possible action of this natural compound against *T. cruzi* sirtuins [17]. Compounds **1**–**6** isolated from the CNSL of *A. occidentale* were then evaluated to verify their potential to inhibit *T. cruzi* sirtuins (Table 1).

**Figure 3:**
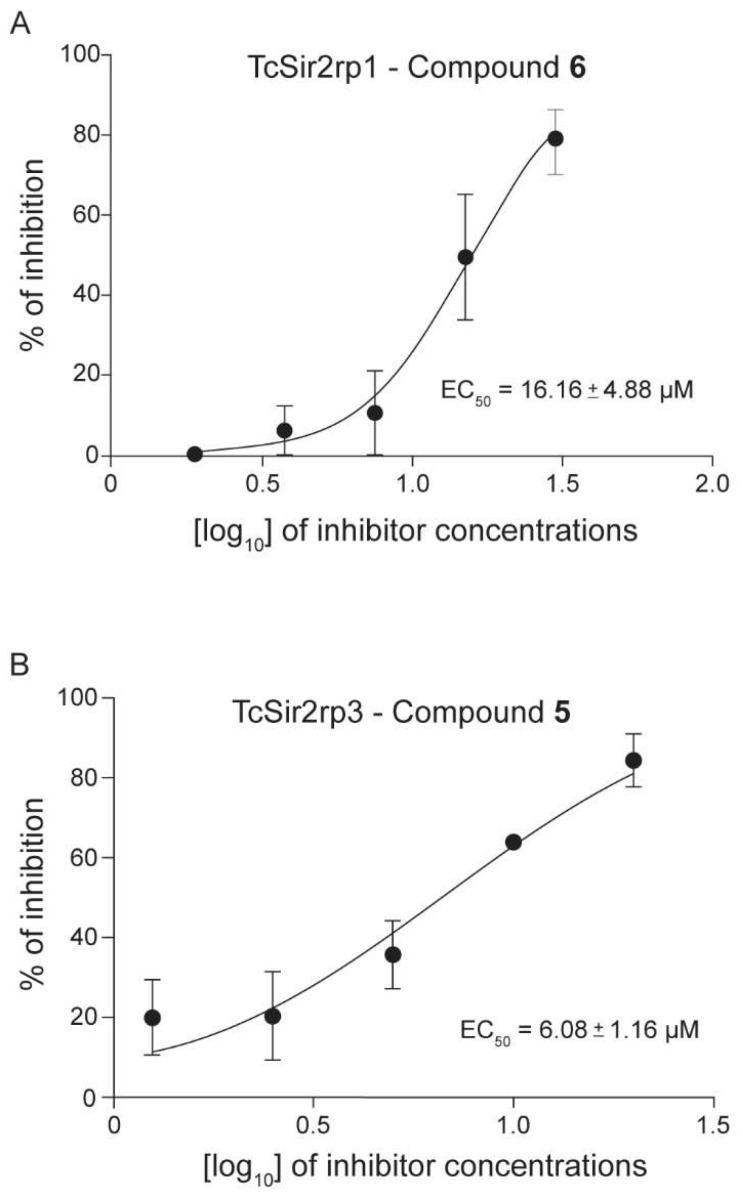
Inhibitory effect of compounds **5** and **6** in TcSir2rp1 and TcSir2rp3 deacetylase activity. Recombinant enzymes were pre-incubated with the compounds at different concentrations with the aim to obtain EC_50_. After 30 min, the deacetylase reactions were initiated by adding the peptide substrate. (**A**) Log concentration-response curve of compound **6** against TcSir2rp1. (**B**) Log concentration-response curve of compound **5** against TcSir2rp3. Similar results were obtained with the compounds that inhibited TcSir2rp1 and/or TcSir2rp3. Bars represent the mean ± standard error of the mean (SEM) of three independent experiments performed.

As shown in Table 1, three compounds inhibited TcSir2rp1, while two inhibited TcSir2rp3. Both anacardic acids presented the best potency against TcSir2rp1 (Compound **6**: EC_50_ = 16.16 µM), and TcSir2rp3 (Compound **5**: EC_50_ = 6.08 µM) (Figure 3). These two compounds inhibited both sirtuins of *T. cruzi*. Compound **2** inhibited only TcSir2rp1 with lesser potency when compared to the others (EC_50_ = 31.40 µM).

### 2.3. Anti-Trypanosoma cruzi In Vitro Evaluations

After identifying the compounds that inhibited deacetylase activity, we tested their anti-parasite activity and mammalian cell toxicity. As shown in Table 1, compound **2**, which only inhibited the TcSir2rp1 enzyme, caused trypomastigote death and inhibition of intracellular amastigotes proliferation. Compound **2** trypomastigote EC_50_ was similar to benznidazole EC_50_ (12.25 µM when compared to 11.40 µM). Compound **2** also showed a selectivity index of 4. This index was based on the effect of the molecule on amastigote growth and mammalian cell toxicity. Compounds **5** and **6**, which affected both sirtuins, inhibited the growth of amastigotes at relatively higher concentrations (EC_50_ ~ 40 µM) with no effect on the trypomastigotes. Interestingly, these compounds were not toxic to human primary skin fibroblasts (hFIB). We also observed that compounds **1** and **4**, even though they did not inhibit sirtuins, presented anti-*T. cruzi* effects against trypomastigotes (23.36 and 43.52 µM, respectively) and amastigotes (11.75 and 15.28 µM, respectively), while compound **3** only inhibited amastigote forms with an EC_50_ of 46.98 µM. These data suggest that those compounds could be targeting different enzymes and act through different mechanisms. Cardol constituents (**1** and **2)** presented the highest activity against the parasite when compared to cardanol substances and anacardic acids. For this reason, we selected compound **2**, a TcSir2rp1 inhibitor, to evaluate its combinatory effect with benznidazole against amastigote forms of parasite through in vitro infection assays. Compared to individual drug treatment (compound **2** or benznidazole), the combination of both compounds reduced only the EC_50_ value of compound **2** (Table 2). Combination index (CI) calculations were used as cutoffs and revealed that this combination did not have a synergistic effect against infected hFIB.

**Figure 4:**
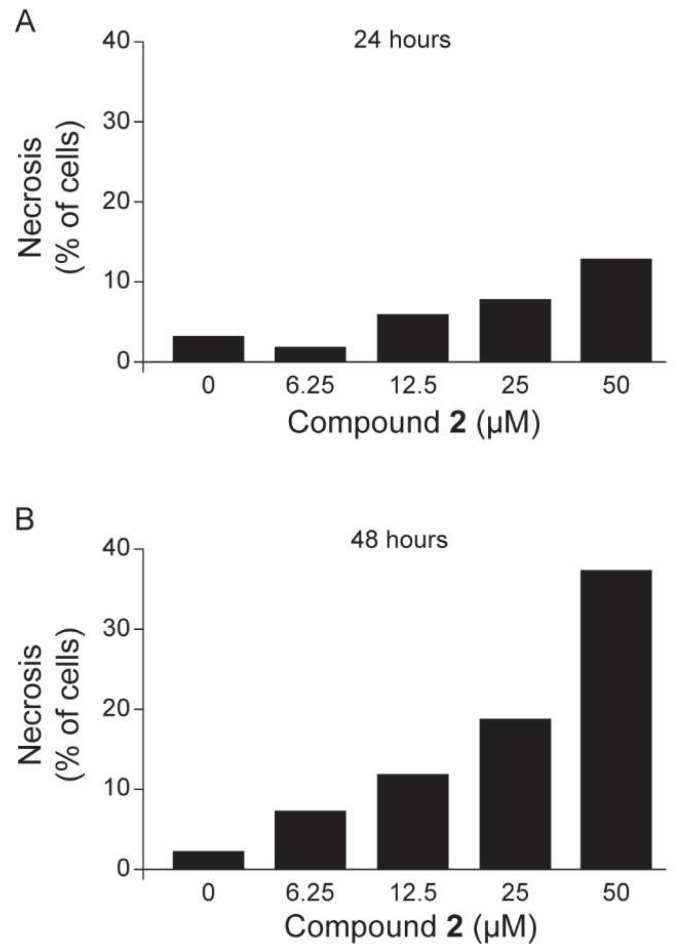
Treatment with compound **2** caused parasite death by inducing necrosis. Trypomastigotes were treated for 24 and 48 h with **2** at 6.25 µM, 12.5 µM, 25 µM, and 50 µM. Parasites were examined by flow cytometry with annexin V and PI staining. (**A**) Relationship between concentration of compound **2** and percentage of necrosis after 24 h treatment. (**B**) Relationship between concentration of compound **2** and percentage of necrosis after 48 h treatment.

As compound **2** had the best activity against the parasites and also inhibited TcSir2rp1, we investigated the mechanism of cell death induced by treatment with this compound in the trypomastigotes. Parasites were incubated with different concentrations of compound **2** for 24 and 48 h at 37 °C, and then double labeled with annexin V-fluorescein isothiocyanate (FITC) and propidium iodide (PI). Individual cellular data were acquired and analyzed by flow cytometry. In comparison to untreated parasites, a concentration-dependent increase in the percentage of stained parasites was observed after compound **2** treatment. Parasites treated with compound **2** at 50 µM for 24 h showed 11.8% PI positive staining, while after 48 h, its staining increased to 37.2%, indicating a necrotic effect post parasite treatment. As shown in Figure 4, this cardol compound (**2**) increased the proportion of necrotic *T. cruzi* cells in a concentration-dependent manner.

## 3. Discussion

Compounds **1**–**6** were first reported in 1986, which were isolated from the hexane extract from cashew nut, and showed interesting molluscicidal activity, which proved to be an interesting approach for preventing schistosomiasis, another parasitic disease [25]. Here, we investigated the effect of these six chemical constituents against the two sirtuins present in *T. cruzi*.

Among the compounds studied, the cardanols (**3** and **4**) did not inhibit *T. cruzi* sirtuins, while one cardol (**2**) inhibited TcSir2rp1, and anacardic acids (**5** and **6**) inhibited both *T. cruzi* sirtuins. In a previous study by our group, a virtual screening of a library of compounds including compounds **5** and **6** suggested the potential of these molecules in inhibiting *T. cruzi* sirtuins [17]. Here, we validated this prediction data, demonstrating that anacardic acids may act as TcSir2rp1 and TcSir2rp3 inhibitors. Anacardic acids have already been described as *T. cruzi* glyceraldehyde-3-phosphate dehydrogenase inhibitors [32], indicating that this class of compounds are an interesting source for the development of anti-*T. cruzi* agents.

Regarding the antiparasitic activity, compound **2** exhibited activity against the trypomastigote and amastigote forms of *T. cruzi*, while compounds **5** and **6** inhibited only the proliferation of the intracellular form of the parasite. Interestingly, compounds **1** and **4** also exhibited antiparasitic effects but were not related to the inhibition of parasite sirtuins, which indicates the presence of off targets.

Different biological properties of CNSL have been described in the literature, mainly associated with its phenolic constituents (anacardic acid, cardol, and cardanol). All of these molecules share a side chain of 15 carbon atoms differing in the degree of unsaturation (monoene, diene, and triene) [22], showing that *A. occidentale* is an important source of phenolic compounds containing an unsaturated long carbon chain at the *meta* position, which are difficult to obtain by synthetic routes [31].

It has recently been demonstrated that the unsaturation of the anacardic acid side-chain plays an important role in determining bioactivity [28]. Regarding the anacardic acids **5** and **6** (diene-15:2, monoene-15:1), no differences in biological activity were observed, suggesting that the position of the double bond is not relevant for TcSir2rp1 and TcSir2rp3 activity. In relation to the compounds classified as cardol **1** and **2** (triene-15:3, diene-15:2), however, differences between the unsaturation in the carbon chain of the molecules resulted in a different TcSir2rp1 enzyme inhibition activity. The cardol diene-15:2 (**2**) inhibited the cytoplasmic sirtuin of the parasite besides inhibiting the viability of the parasite, while the cardol triene-15:3 (**1**) had no inhibitory activity.

Although we found that compounds **1**–**6** presented trypanocidal activity, they did not present good selectivity. Drug combination therapies are of interest due to the possible synergistic effect and reduced toxicity effect of the treatment. Benznidazole is known to exert activity through the induction of reductive stress, involving the covalent modification of cellular macromolecules by intracellular reduced metabolites of the parent compound by an action of nitroreductases [33]. Considering that compound **2** and benznidazole exhibit different mechanisms of antiparasitic actions, the possibility of drug combination was studied. The antiparasitic assay, however, did not show any synergistic effects with these two compounds.

Our results showed the potential of *A. occidentale* as a natural source of TcSir2rp1 and TcSir2rp3 inhibitors and anti-*T. cruzi* agents. These biological activities encourage further investigations to look for optimized structures with reduced cytotoxicity as possible drug candidates for the treatment of Chagas disease.

## 4. Materials and Methods

### 4.1. General Experimental Procedures

NMR spectroscopic data were recorded on a 500 MHz Varian (Palo Alto, CA, USA) INOVA NMR spectrometer. Complete assignments were obtained based on 2D-NMR experiments (COSY, NOESY, HSQC, and HMBC). HRESIMS was performed on a Waters Acquity UPLC system coupled to a Waters Micromass LCT Premier Time-of-Flight mass spectrometer (Milford, MA, USA) equipped with an electrospray interface (ESI). HPLC-PDA-ESIMS analysis was conducted on an HP 1200 system equipped with a photodiode array detector (Agilent Technologies, Santa Clara, CA, USA) connected to a Finnigan MAT LCQ ion-trap mass spectrometer (Finnigan, San Jose, CA, USA), equipped with a Finnigan electrospray interface (ESI). MPLC was performed using a Büchi system equipped with a C-660 module pump, C-640 UV detector, C-684 fraction collector and the system was controlled by the software Sepacore Control (version SepacoreControl 1.3.3000.4091 Standard Edition, Büchi, Flawil, Switzerland).

### 4.2. Plant Material

The cashew nuts of *A. occidentale* were collected from Costa Abajo, Santa Rosa, Río Indio on 18 April 1997. The plant was identified by Emeritus Professor Mireya Correa, University of Panama and has been deposited in the Herbarium of the University of Panama (PMA) (Voucher specimen FLORPAN: no. 2894).

### 4.3. Extraction

The cashew nuts (513 g) of *A. occidentale* were cut in halves and extracted with organic solvents of increasing polarity: *n*-hexane, CH_2_Cl_2_, and MeOH. The extracts were concentrated to dryness by rotatory evaporation at <40 °C yielding: *n*-hexane (86.2 g), CH_2_Cl_2_ (29.0 g), and MeOH (37.3 g).

### 4.4. Purification of the Dichloromethane Extract of Cashew Using MPLC and Semi-Preparative HPLC

The CH_2_Cl_2_ extract (3.37 g) was fractionated by MPLC using Zeoprep C_18_ as the stationary phase (15–25 μm, 920 × 49 mm i.d., Zeochem) and an acidic (0.1% formic acid) H_2_O (A) ACN (B) gradient: 70% B in 52.1 h, 70 to 80% B in 3.2 h, 80% B in 32.2 h, 80 to 100% B in 3.2 h, and 100% B for 6.4 h. These conditions were optimized on an HPLC column (15–25 μm, 250 × 4.6 mm i.d., Zeochem, Uetikon am See, Switzerland) packed with the same stationary phase. The extract, prepared by mixing 3.37 g of the CH_2_Cl_2_ extract with 10 g of the Zeoprep C_18_ stationary phase, was introduced into the MPLC column by dry injection. The mixture was conditioned in a dry-load cell (11.5 × 2.7 cm i.d.). The dry-load cell was connected subsequently between the pumps and the MPLC column. The flow rate was set to 5 mL/min, and UV detection was performed at 280 nm. The MPLC separation yielded 263 fractions, which were analyzed by UHPLC-TOF-HRESIMS in order to localize the anarcadic acids, cardols, and cardanols. Using this strategy, it was possible to identify fractions 29 to 31 as containing a pure cardol (**1**), fractions 46 to 51 as containing another pure cardol (**2**), and fractions 210 to 212 as containing a pure anarcadic acid (**6**). Fractions 112 to 117, 124 to 131, and 273 to 288 were selected for purification by reverse-phase semi-preparative HPLC-UV using a column X-Bridge C_18_ (5 μm, 19 × 150 mm i.d.; Waters) with an acidic (0.1% formic acid) H_2_O (A) ACN (B) as the solvent system for a linear gradient elution. The flow rate was set to 10 mL/min, and UV absorbance was detected at 254, 280, and 254 nm, respectively. Fractions 112 to 117 (81.8 mg) were combined and purified by an isocratic elution (70% ACN in 50 min) and led to the isolation of cardanol **3** (39.3 mg). Fractions 124 to 131 (77.3 mg) were also combined and purified by an isocratic elution (75% ACN in 40 min) and afforded anarcadic acid **5** (20.1 mg). Fractions 273 to 288 (64.5 mg) were combined and purified by an isocratic elution (70% ACN in 60 min) and yielded cardanol **4** (45.5 mg).

### 4.5. Recombinant TcSir2rp1 and TcSir2rp3

The TcSir2rp1 gene was amplified from the genomic DNA of the *T. cruzi* Y strain using the oligonucleotides TcSir2rp1_NheI_F_5′-GCTAGCCATATGAATCAAGATAACGCCAACT-3′ and TcSir2rp1_XhoI_R_5′-CCCTCGAGTTATTTTCGGTCTGT-3′, inserted in the respective sites of pET28a (Novagen, Itapira, Brazil) and used to transform BL21 (DE3) bacteria (Agilent Technologies, Santa Clara, CA, USA). The protein was obtained by induction with 1 mM isopropyl β-d-1-thiogalactopyranoside (IPTG) at 37 °C for 2 h. Bacterial cells were collected by centrifugation (3.600× *g* for 10 min) and the pellet was resuspended in cold lysis buffer (200 mM NaCl, 5% glycerol, 5 mM 2-mercaptoethanol, and 25 mM HEPES-NaOH [pH 7.5] and protease inhibitor cocktails) (Sigma-Aldrich, St. Louis, MO, USA). The bacterial cells were lysed by three passages on a French press apparatus (Thermo Electron Corporation, Beverly, MA, USA) and the lysate was clarified by centrifugation and incubated for 1 h at 4 °C with nickel nitrilotriacetic acid (Ni-NTA) Superflow beads (Qiagen, Gaithersburg, MD, USA). The resin was washed with lysis buffer containing 20 mM imidazole, the enzyme was eluted with one volume of the same buffer containing 0.25 M imidazole, and stored at −80 °C.

Recombinant TcSir2rp3 was obtained as described previously by [8]. The TcSir2rp3 gene was amplified from the genomic DNA of the *T. cruzi* Y strain using the oligonucleotides TcSir2rp3_BamHI_F_5′-CTGGATCCATGAGGCCGCGGCGTC AGG-3′ and TcSir2rp3_XhoI_R_5′-CTCGAGCTACACCGCGTCTTGAAGGAGCT-3′ and cloned into pET28a (Novagen, Itapira, Brazil). The recombinant vector was used to transform ArcticExpress DE3 bacteria (Agilent Technologies) and protein was expressed by induction with 0.1 mM IPTG at 12 °C.

### 4.6. Deacetylase Activity Assay

Deacetylase assays were performed from the protocol established previously by [8]. In summary, the reaction was performed in two steps. In the first step, a synthetic peptide as the substrate (Abz-Gly-Pro-acetyl-Lys-Ser-Gln-EDDnp, where Abz is ortho-aminobenzoic acid and EDDnp is *N*-[2,4-dinitrophenyl]ethylenediamine) was dissolved in 50 µL of 25 mM Tris-HCl (pH 8.0), 137 mM NaCl, 2.7 mM KCl, and 1 mM MgCl_2_ containing 0.6 mM NAD^+^ (Sigma-Aldrich). TcSir2rp1 or TcSir2rp3 were added and the reactions performed for 4 h at 30 °C. In the inhibition experiments, the enzyme was pre-treated for 30 min at room temperature with the indicated compounds and the addition of the substrate started the reaction. In the second step, the reactions were stopped with 50 µL of a solution containing 12 mM nicotinamide in 100 mM NaCl and 50 mM Tris-HCl (pH 8.0), in the absence or presence of 0.6 mg/mL trypsin (Sigma-Aldrich). After further incubation for 15 min, the fluorescence at 420 nm was read (excitation, 320 nm) in a SpectraMax M3 plate reader (Molecular Devices, Sunnyvale, CA, USA). The percentage of deacetylase activity was determined in comparison with the negative control, and inhibitions of TcSir2rp1 and TcSir2rp3 by the treatment were expressed as the 50% effective concentration (EC_50_).

### 4.7. Host Cell Toxicity

The procedures were approved by the local Ethics Committee (Hospital São Rafael, CAAE: 20032313.6.0000.0048). Human primary skin fibroblasts (hFIB) were obtained from patient skin biopsy and cultured in Dulbecco’s Modified Eagle Medium (DMEM) supplemented with 10% fetal bovine serum (FBS) and Penicillin-Streptomycin (0.2 U/mL) (all from Gibco/Life Technologies, Grand Island, NY, USA). The cells were seeded on 96-well plates at 3 × 10^4^ cells/mL and treated with the drugs for 72 h of incubation time. Following the treatments, cells were washed with saline solution twice, and cell viability was determined by the AlamarBlue assay (Thermo Fisher Scientific, Carlsbad, CA, USA), according to the manufacturer’s instructions. Colorimetric readings were performed after 24 h at 570 and 600 nm. Fifty-percent cytotoxic concentration (CC_50_) values were calculated using data points gathered from three independent experiments.

### 4.8. Parasites

All experiments were performed with the Y strain of *T. cruzi*. Trypomastigote forms of *T. cruzi* were obtained from the supernatant of infected LLC-MK2 cells and maintained in DMEM supplemented with 10% FBS and Penicillin-Streptomycin (100 U/mL) (all from Gibco/Life Technologies) at 37 °C with 5% CO_2_.

### 4.9. Activity Against Trypomastigotes

Trypomastigotes were incubated in 96-well plates (2 × 10^6^ cells/mL) in DMEM, in the presence or absence of the compounds at different concentrations for 24 h. Viable parasites (refringent and moving cells) were counted in a hemocytometer, and compound antiparasitic activity was expressed as 50% effective concentration (EC_50_), in comparison to the untreated parasites. Each compound concentration was tested in triplicate, and three independent experiments were performed. The reference drug, benznidazole, was used as the positive control.

### 4.10. In Vitro T. cruzi Infection Assay

hFIB (3 × 10^4^ cells/mL) cultured in DMEM was seeded on 96-well plates at 3 × 10^4^ cells/mL and maintained overnight at 37 °C with 5% CO_2_. The culture was washed with saline solution and infected with trypomastigotes (10 parasites:1 host cell). Following 24 h of incubation, the non-internalized parasites were removed by washing with saline solution, and fresh medium, with or without compounds at different concentrations, was added to the culture before the incubations proceeded for 72 h. Cells were then fixed and stained by the addition of 50 μL of a solution containing 4% paraformaldehyde and 4 μM Draq5 DNA dye (BioStatus, Shepshed, UK) per well. Plates were imaged in an Operetta High Content Imaging System (PerkinElmer, Waltham, MA, USA) confocal microscope using a 10X air objective. Nine images were collected from each well for reliable statistical analysis. The percentage of intracellular parasites was determined and compared to the negative control, and compound antiparasitic activity was also expressed as EC_50_. The reference drug, benznidazole, was used as the positive control at 50 µM. Each drug concentration was carried out in triplicate. For in vitro drug combination, doubling dilutions of each drug (compound **2** and benznidazole) used alone or in fixed combinations were incubated for 72 h after hFIB infection, as described above. The analysis of the combined effects was performed by determining the combination index (CI) and was used as the cutoff to determine synergism, additivity, or antagonism by using the Chou–Talalay CI method [34].

### 4.11. Flow Cytometry Analysis

Trypomastigotes (10^7^ cells/mL) were resuspended in supplemented DMEM medium and treated with compound **2** (50, 25, 12.5, and 6.25 µM) for 24 and 48 h at 37 °C with 5% CO_2_. Parasites were labeled with propidium iodide (PI) and annexin V using the annexin V-fluorescein isothiocyanate (FITC) apoptosis detection kit (BD Biosciences, San Jose, CA, USA), according to the manufacturer’s instructions. The experiment was performed using a BD LSR Fortessa SORP (San Jose, CA, USA) by acquiring 10,000 events and the data were analyzed by FlowJo VX (Ashland, OR, USA).

### 4.12. Statistical Analyses

Nonlinear regression analysis was used to calculate the EC_50_ and CC_50_ values. The selectivity index (SI) was defined as the ratio of CC_50_ (hFIB) to EC_50_ (amastigote form). One-way ANOVA followed by Bonferroni’s multiple comparison test was used to determine the statistical significance of the group comparisons in the in vitro NAD^+^-deacetylase activity of TcSir2rp1 and TcSir2rp3 recombinant proteins. Results were considered statistically significant when *p* < 0.05. Analyses were performed using GraphPad Prism version 5.01 (Graph Pad Software, San Diego, CA, USA).

## Figures and Tables

**Table 1:** Deacetylase (TcSir2rp1 and TcSir2rp3) and antiparasitic activity in trypomastigote, intracellular parasite, host cell cytotoxicity, and selectivity index of *Anacardium occidentale* chemical compounds.

Compounds	TcSir2rp1	TcSir2rp3	Trypomastigote	Amastigote	hFIB	SI ^e^
	EC_50_ ± SEM (µM) ^a^	EC_50_ ± SEM (µM) ^a^	EC_50_ ± SEM (µM) ^b^	EC_50_ ± SEM (µM) ^c^	CC_50_ ± SEM (µM) ^d^	
**1**	NA	NA	23.36 ± 0.12	11.75 ± 0.40	47.17 ± 0.10	4
**2**	31.40 ± 2.33	NA	12.25 ± 0.25	14.70 ± 3.27	53.70 ± 7.08	4
**3**	NA	NA	NA	46.98 ± 1.97	>100	>2
**4**	NA	NA	43.52 ± 2.32	15.28 ± 2.00	45.47 ± 0.27	3
**5**	23.35 ± 2.51	6.08 ± 1.16	NA	41.67 ± 5.53	>100	>2
**6**	16.16 ± 4.88	7.41 ± 0.65	NA	42.39 ± 5.11	>100	>2
Benznidazole			11.40 ± 1.09	3.13 ± 0.87	>100	>32

^a^ Sirtuin activity was determined 4 h after incubation. ^b^ Determined 24 h after incubation with compounds. ^c^ Infected human primary skin fibroblasts (hFIB) were exposed to compounds and activity was determined 72 h after treatment. ^d^ hFIB viability was determined 72 h after treatment. ^e^ Selectivity index (SI), calculated by the ratio of CC_50_ (hFIB) to EC_50_ (amastigote). EC_50_, effective concentration at 50%; CC_50_, cytotoxic concentration at 50%. ND, not determined due to lack of activity. Values were calculated using concentrations in triplicate, and three independent experiments were performed.

**Table 2:** Combined treatment of infected hFIB with compound **2** and benznidazole.

	EC50 ± SD (µM)		
Compounds	Alone	Combination	CI ± SD (µM)
**2**	12.15 ± 6.46	2.69 ± 1.13	1.35 ± 0.04
Benznidazole	2.17 ± 0.09	2.45 ± 0.34	

Cutoff: CI value of 0.30 to 0.70, synergism; 0.70 to 0.85, moderate synergism; 0.85 to 0.90, slight synergism; 0.90 to 1.10, additivity; >1.10, antagonism. CI, combination index; SD, standard deviation.

## Supplementary Materials

The supplementary materials are available online.
