# Supplementary material for: Chemical Constituents of Anacardium occidentale as Inhibitors of Trypanosoma cruzi Sirtuins
Source: Molecules. 2019 Apr 3;24(7):1299. doi: 10.3390/molecules24071299 (PMC6479711; doi:10.3390/molecules24071299)
Supplement: Supplementary file 1 [file molecules-24-01299-s001.pdf]

Supplementary data

Chemical Constituents of *Anacardium occidentale* as Inhibitors of *Trypanosoma cruzi* Sirtuins

Tanira Matutino Bastos [1], Helena Mannochio Russo [2], Nilmar Silvio Moretti [3], Sergio Schenkman [3], Laurence Marcourt [2], Mahabir Prashad Gupta [4], Jean-Luc Wolfender [2], Emerson Ferreira Queiroz [2] and Milena Botelho Pereira Soares [1,*]

[1] Instituto Gonçalo Moniz, FIOCRUZ, Salvador, BA, 40296-710, Brazil; tancomb@hotmail.com (T.M.B.)
[2] School of Pharmaceutical Sciences, EPGL, University of Geneva, University of Lausanne, CMU, 1, Rue Michel Servet, 1211 Geneva, Switzerland; helenamrusso@gmail.com (H.M.R.); laurence.marcourt@unige.ch (L.M.); jean-luc.wolfender@unige.ch (J.-L.W.); emerson.ferreira@unige.ch (E.F.Q.)
[3] Departmento de Microbiologia, Imunologia e Parasitologia, UNIFESP, São Paulo, SP, 04039-032, Brazil; nilmar.moretti@unifesp.br (N.S.M.); sergioschenkman@gmail.com (S.S.)
[4] Center for Pharmacognostic Research on Panamanian Flora (CIFLORPAN), College of Pharmacy, University of Panama, Panama City, 0824-00172, Panama; mahabirgupta@gmail.com (M.P.G.)
* Correspondence: milena@bahia.fiocruz.br; Tel.: +55 (71) 3176-2272.

1. Description of the isolated compounds

*1.1. Compound **1***

Amorphous solid, ^{1}H NMR (DMSO-d_6, 500 MHz) δ 1.28 (8H, m, H-3, H-4, H-5, H-6), 1.48 (2H, p, *J* = 7.2 Hz, H-2), 2.01 (2H, q, *J* = 6.7 Hz, H-7), 2.35 (2H, t, *J* = 7.2 Hz, H-1), 2.75 (2H, t, *J* = 6.0 Hz, H-10), 2.80 (2H, t, J = 6.0 Hz, H-13), 4.96 (1H, dq, *J* = 10.1, 1.7 Hz, H-15cis), 5.03 (1H, dq, *J* = 17.1, 1.7 Hz, H-15trans), 5.30 (1H, m, H-9), 5.33 (1H, m, H-8), 5.38 (2H, m, H-11, H-12), 5.79 (1H, ddt, *J* = 17.1, 10.1, 6.0 Hz, H-14), 6.01 (3H, s, H-2', H-4', H-6'); 13C NMR (DMSO-d_6, 126 MHz) δ 25.1 (C-10), 26.6 (C-7), 28.6, 28.6, 28.7, 29.0 (C-3, C-4, C-5, C-6), 30.7 (C-2), 31.0 (C-13), 35.3 (C-1), 99.9 (C-4'), 106.3 (C-2', C-6'), 114.8 (C-15), 126.5 (C-12), 127.4 (C-9), 128.9 (C-11), 129.9 (C-8), 136.7 (C-14), 144.1 (C-1'), 158.1 (C-3', C-5'); HREIMS: *m/z* 313.2171 [M-H]$^-$ (calcd for $C_{21}H_{29}O_2$, 313.2168; Δ ppm = 1.0).

*1.2. Compound **2***

Amorphous solid, ^{1}H NMR (DMSO-d_6, 500 MHz) δ 0.86 (3H, t, *J* = 7.4 Hz, H-15), 1.30 (10H, m, H-3, H-4, H-5, H-6, H-14), 1.48 (2H, p, *J* = 7.0 Hz, H-2), 2.01 (4H, m, H-7, H-13), 2.35 (2H, t, *J* = 7.6 Hz, H-1), 2.74 (2H, t, *J* = 6.2 Hz, H-10), 5.32 (4H, m, H-8, H-9, H-11, H-12), 6.01 (3H, s, H-2', H-4', H-6'); ^{13}C NMR (DMSO-d_6, 126 MHz) δ 13.5 (C-15), 22.2 (C-14), 25.2 (C-10), 26.6 (C-7), 28.6 (C-13), 28.7, 28.7, 28.7, 29.0 (C-3, C-4, C-5, C-6), 30.7 (C-2), 35.3 (C-1), 99.9 (C-4'), 106.2 (C-2', C-6'), 127.7 (C-11), 127.9 (C-9), 129.4 (C-12), 129.7 (C-8), 144.1 (C-1'), 158.1 (C-3', C-5'); HREIMS *m/z* 317.2471 [M+H]$^+$ (calcd for $C_{21}H_{33}O_2$, 317.2481; Δ ppm = -3.2).

*1.3. Compound **3***

Amorphous solid, ^{1}H NMR (DMSO-d_6, 500 MHz) δ 1.27 (8H, m, H-3, H-4, H-5, H-6), 1.51 (2H, p, *J* = 7.3 Hz, H-2), 2.01 (2H, q, *J* = 6.8 Hz, H-7), 2.46 (2H, t, *J* = 7.3 Hz, H-1), 2.75 (2H, t, *J* = 6.1 Hz, H-10), 2.79 (2H, t, *J* = 6.0 Hz, H-13), 4.96 (1H, dq, *J* = 10.1, 1.7 Hz, H-15cis), 5.03 (1H, dq, *J* = 17.1, 1.9 Hz, H-15trans), 5.35 (4H, m, H-8, H-9, H-11, H-12), 5.79 (1H, ddt, *J* = 17.1, 10.1, 6.0 Hz, H-14), 6.56 (3H, m, H-2', H-4', H-6'), 7.03 (1H, t, *J* = 7.6 Hz, H-3'), 9.20 (1H, s, OH); ^{13}C NMR (DMSO-d_6, 126 MHz) δ 25.1 (C-10), 26.5 (C-7), 28.5, 28.6, 28.9 (C-4, C-5, C-6), 28.6 (C-3), 30.8 (C-2), 31.0 (C-13), 35.1 (C-1), 112.5 (C-4'), 114.8 (C-15), 115.1 (C-6'), 118.8 (C-2'), 126.5 (C-11 or C-12), 127.4 (C-9), 128.9 (C-11 or C-12), 129.0 (C-3'), 129.9 (C-8), 136.7 (C-14), 143.6 (C-1'), 157.2 (C-5'); HREIMS m/z 297.2223 [M-H]$^-$ (calcd for $C_{21}H_{29}O$, 297.2218; Δ ppm = 1.7).

1.4. Compound ***4***

Amorphous solid, ^{1}H NMR (DMSO-d_6, 500 MHz) δ 0.85 (3H, t, *J* = 6.6 Hz, H-15), 1.25 (16H, m, H-3, H-4, H-5, H-6, H-11, H-12, H-13, H-14), 1.51 (2H, p, *J* = 7.5 Hz, H-2), 1.97 (4H, m, H-7, H-10), 2.45 (2H, t, *J* = 7.5 Hz, H-1), 5.31 (2H, m, H-8, H-9), 6.56 (3H, m, H-2', H-4', H-6'), 7.02 (1H, t, *J* = 7.7 Hz, 5'), 9.20 (1H, s, OH); 13C NMR (DMSO-d_6, 126 MHz) δ 13.8 (C-15), 22.0 (C-14), 26.5 (C-10), 26.6 (C-7), 28.3, 28.5, 28.6, 28.7, 29.1 (C-3, C-4, C-5, C-6, C-11, C-12), 30.8 (C-2), 31.1 (C-13), 35.1 (C-1), 112.5 (C-4'), 115.1 (C-2'), 118.7 (C-6'), 128.9 (C-5'), 129.5 (C-8, C-9), 143.6 (C-1'), 157.2 (C-3'); HREIMS *m*/*z* 301.2538 [M-H]$^-$ (calcd for $C_{21}H_{33}O$, 301.2531; Δ ppm = 2.3).

1.5. Compound ***5***

Amorphous solid, ^{1}H NMR (DMSO-d_6, 500 MHz) δ 0.86 (3H, t, *J* = 7.4 Hz, H-15), 1.32 (10H, m, H-3, H-4, H-5, H-6, H-14), 1.50 (2H, p, *J* = 7.6 Hz, H-2), 2.00 (4H, m, H-7, H-13), 2.61 (2H, t, *J* = 7.6 Hz, H-1), 2.74 (2H, t, *J* = 6.0 Hz, H-10), 5.32 (4H, m, H-8, H-9, H-11, H-12), 6.65 (1H, dd, *J* = 7.8, 1.1 Hz, H-2'), 6.69 (1H, dd, *J* = 7.8, 1.1 Hz, H-4'), 7.14 (1H, t, *J* = 7.8 Hz, H-3'), 11.73 (1H, s, COOH); 13C NMR (DMSO-d_6, 126 MHz) δ 13.5 (C-15), 22.2 (C-14), 25.2 (C-10), 26.6 (C-7), 28.6, 28.6, 29.0, 29.0 (C-3, C-4, C-5, C-6), 28.7 (C-13),31.0 (C-2), 33.6 (C-1), 113.5 (C-4'), 119.9 (C-2'), 120.3 (C-6'), 127.7 (C-9 or C-11), 127.9 (C-9 or C-11), 129.4 (C-12), 129.7 (C-8), 130.4 (C-3'), 141.6 (C-1'), 156.3 (C-5'), 170.4 (C-7'); HREIMS *m*/*z* 343.2278 [M-H]$^-$ (calcd for $C_{22}H_{31}O_3$, 343.2273; Δ ppm = 1.5).

1.6. Compound ***6***

Amorphous solid, ^{1}H NMR (DMSO-d_6, 500 MHz) δ 0.84 (3H, t, *J* = 6.9 Hz, H-15), 1.26 (16H, m, H-3, H-4, H-5, H-6, H-11, H-12, H-13, H-14), 1.50 (2H, p, *J* = 7.6 Hz, H-2), 1.97 (4H, m, H-7, H-10), 2.61 (2H, t, *J* = 7.6 Hz, H-1), 5.31 (2H, m, H-8, H-9), 6.65 (1H, d, *J* = 8.0 Hz, H-2'), 6.70 (1H, d, *J* = 8.0 Hz, H-4'), 7.14 (1H, t, *J* = 8.0 Hz, H-3'); ^{13}C NMR (DMSO-d_6, 126 MHz) δ 13.9 (C-15), 22.1 (C-14), 26.6 (C-7, C-10), 28.3, 28.6, 28.7, 29.0, 29.1 (C-3, C-4, C-5, C-6, C-11, C-12), 31.0 (C-2), 31.1 (C-13), 33.6 (C-1), 113.5 (C-4'), 120.0 (C-2'), 120.2 (C-6'), 129.6 (C-8, C-9), 130.5 (C-3'), 141.7 (C-1'), 156.3 (C-5'), 170.4 (C-7'); HREIMS *m*/*z* 345.2435 [M-H]$^-$ (calcd for $C_{22}H_{33}O_3$, 345.2430; Δ ppm = 1.4).

Supporting Information

Figures S1 to S31 showing the HPLC-UV chromatogram and NMR spectra of compounds **1**-**6**.

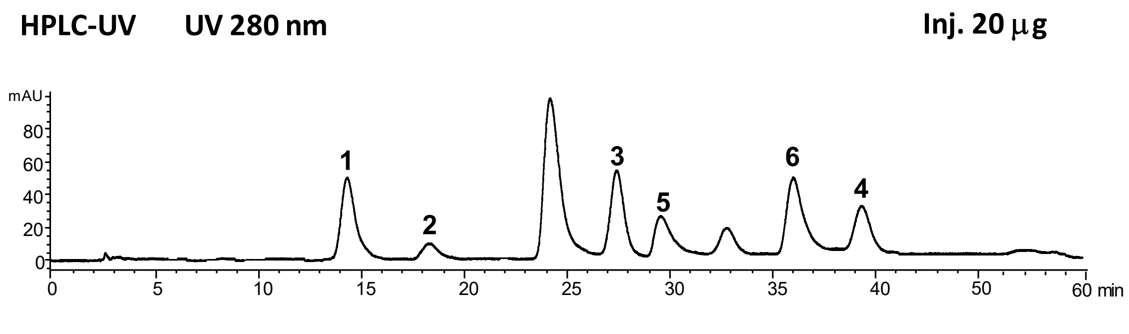

Figure 1S. HPLC-UV analysis (280 nm) of the cashew nut (*Anacardium occidentale*) dichloromethane extract.

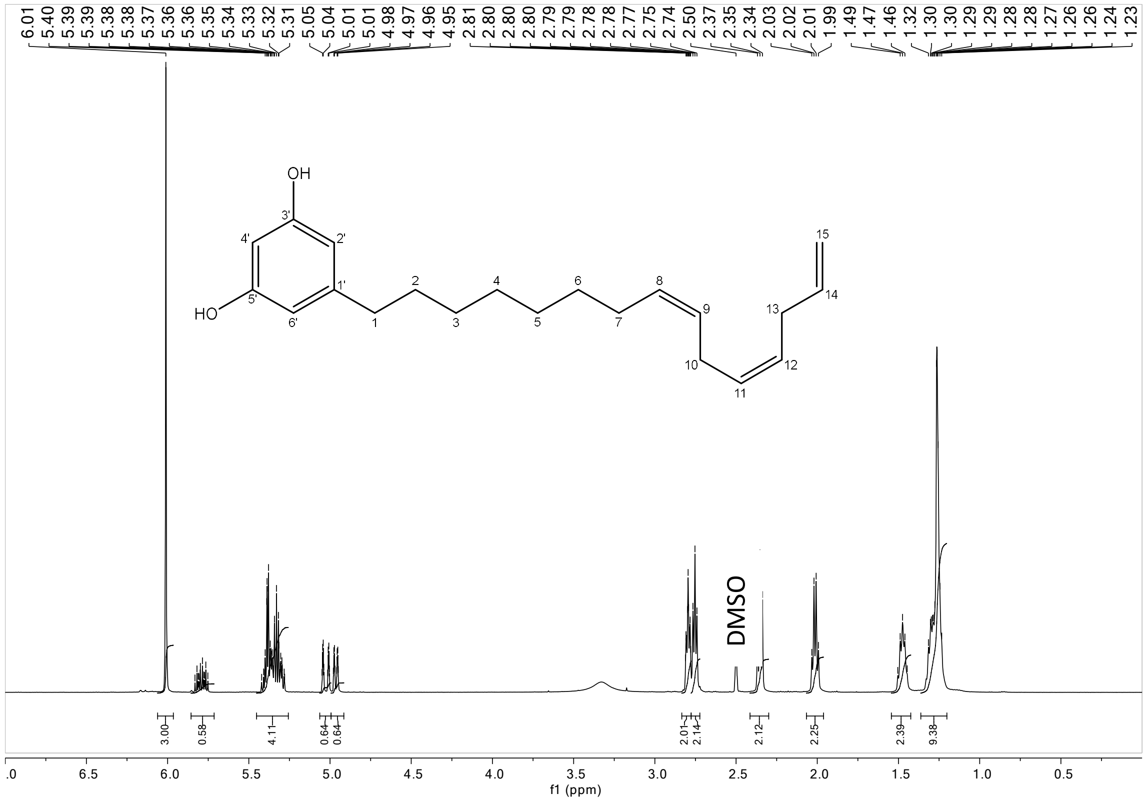

Figure 2S. ^{1}H NMR spectrum of compound **1** in DMSO-d_6.

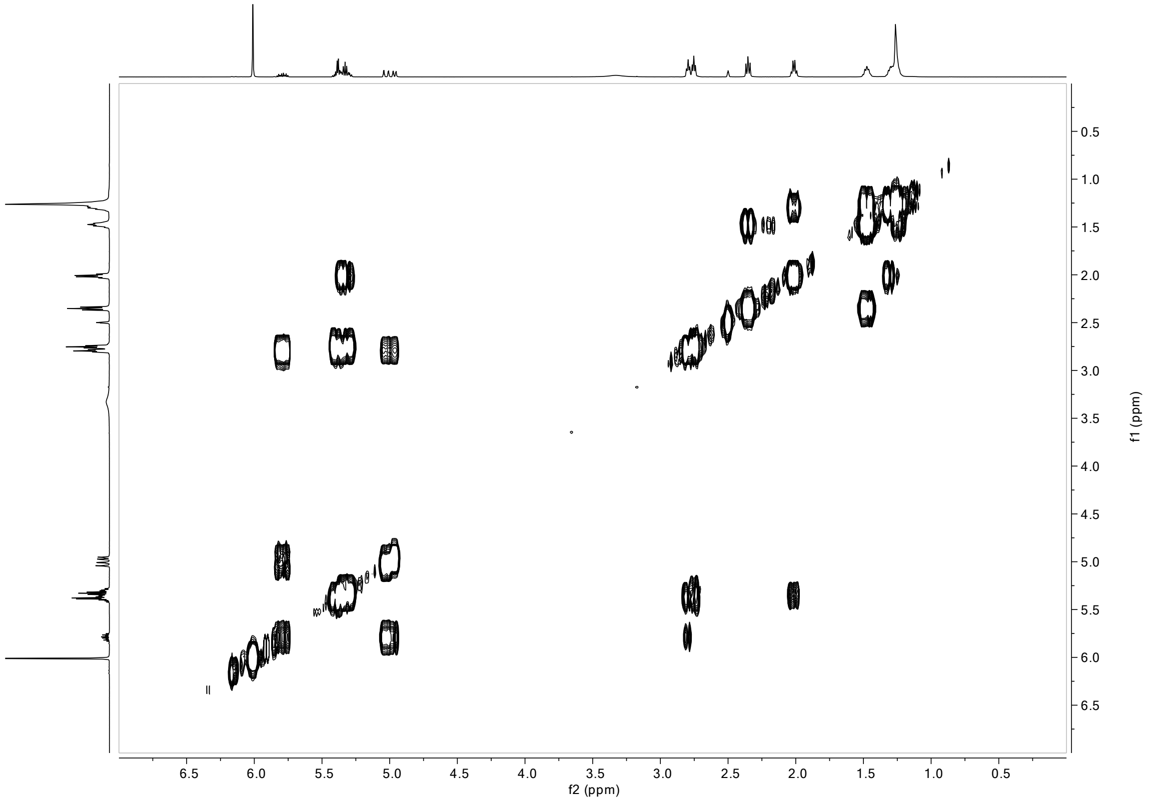

Figure 3S. COSY NMR spectrum of compound **1** in DMSO-*d*₆.

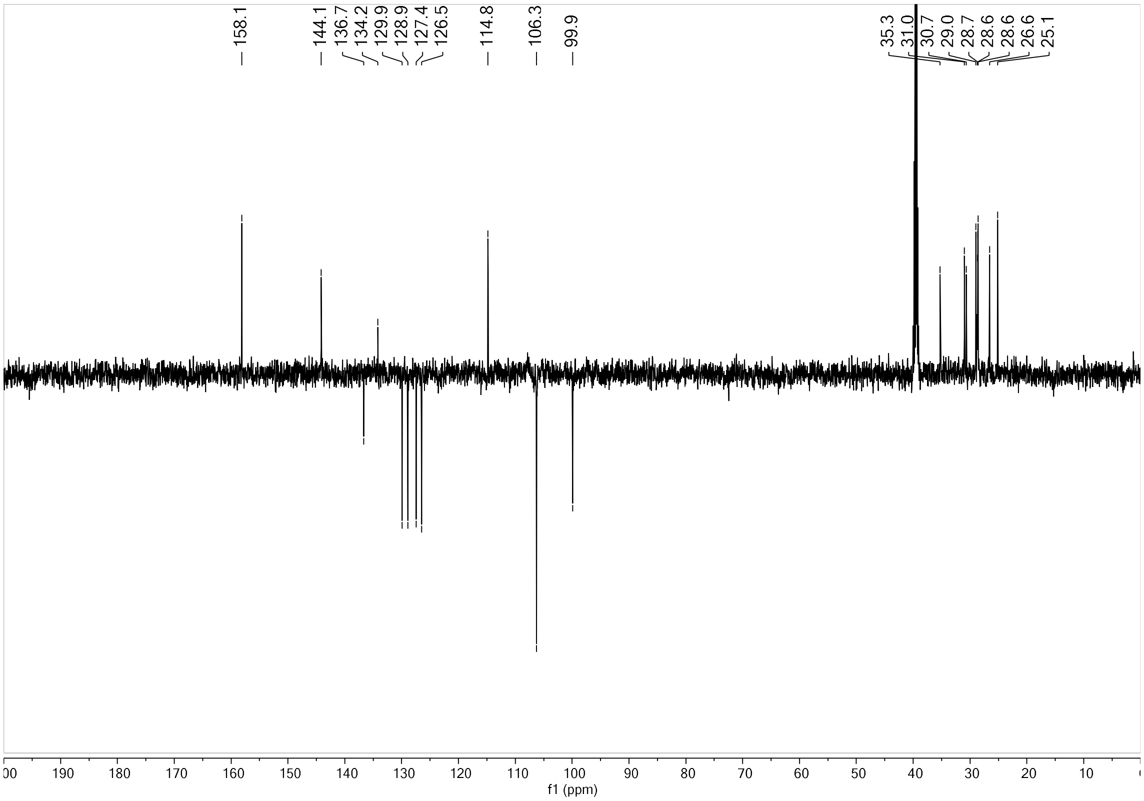

Figure 4S. ^{13}C NMR spectrum of compound **1** in DMSO-*d*₆.

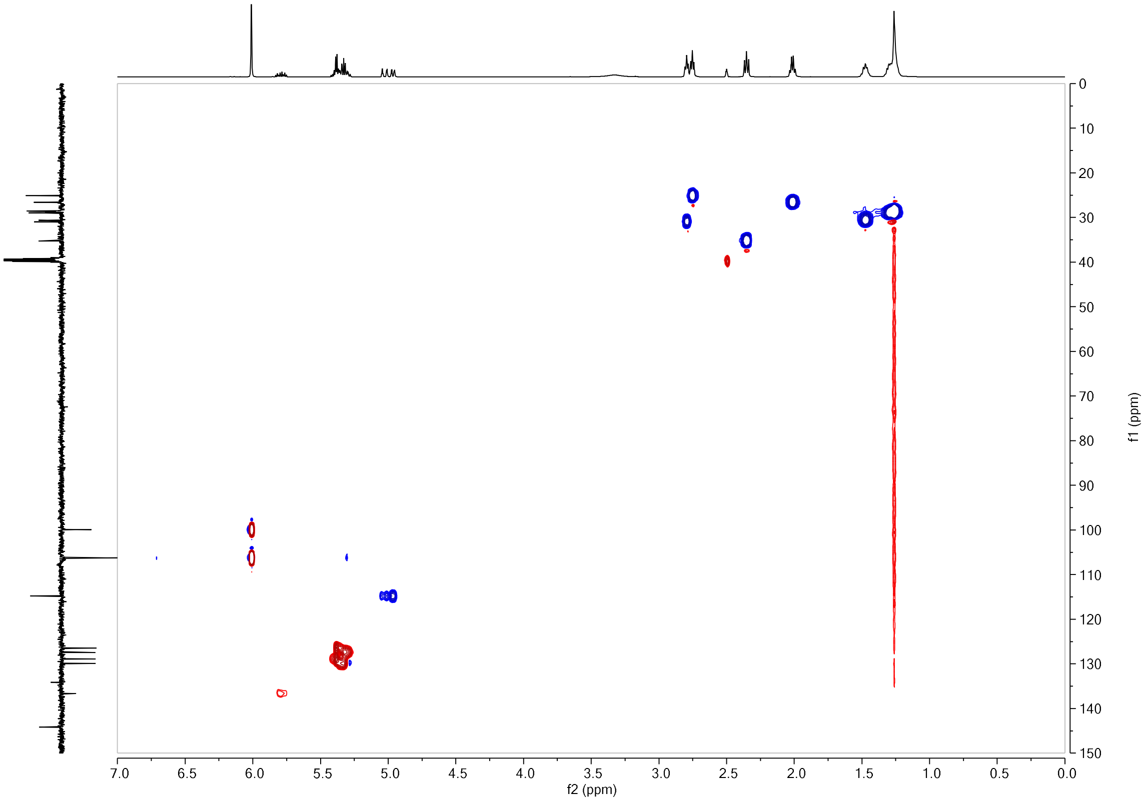

Figure 5S. Edited-HSQC NMR spectrum of compound **1** in DMSO-d_6.

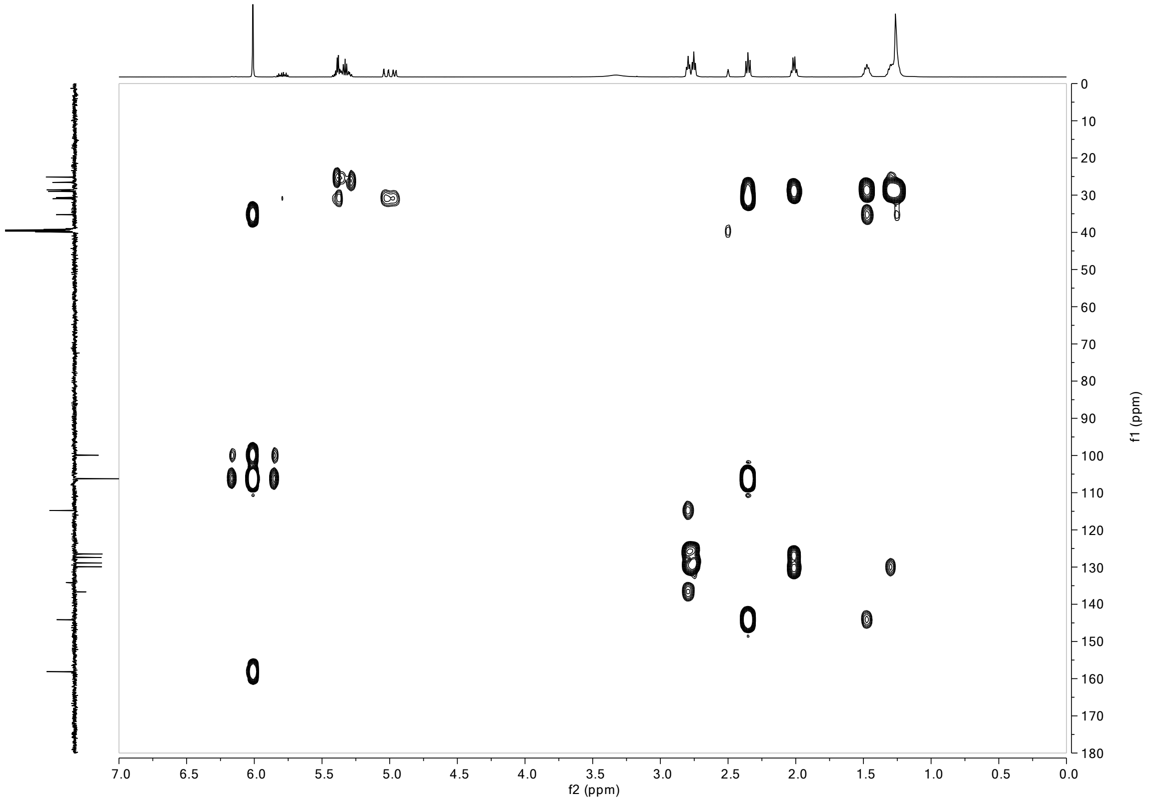

Figure 6S. HMBC NMR spectrum of compound **1** in DMSO-d_6.

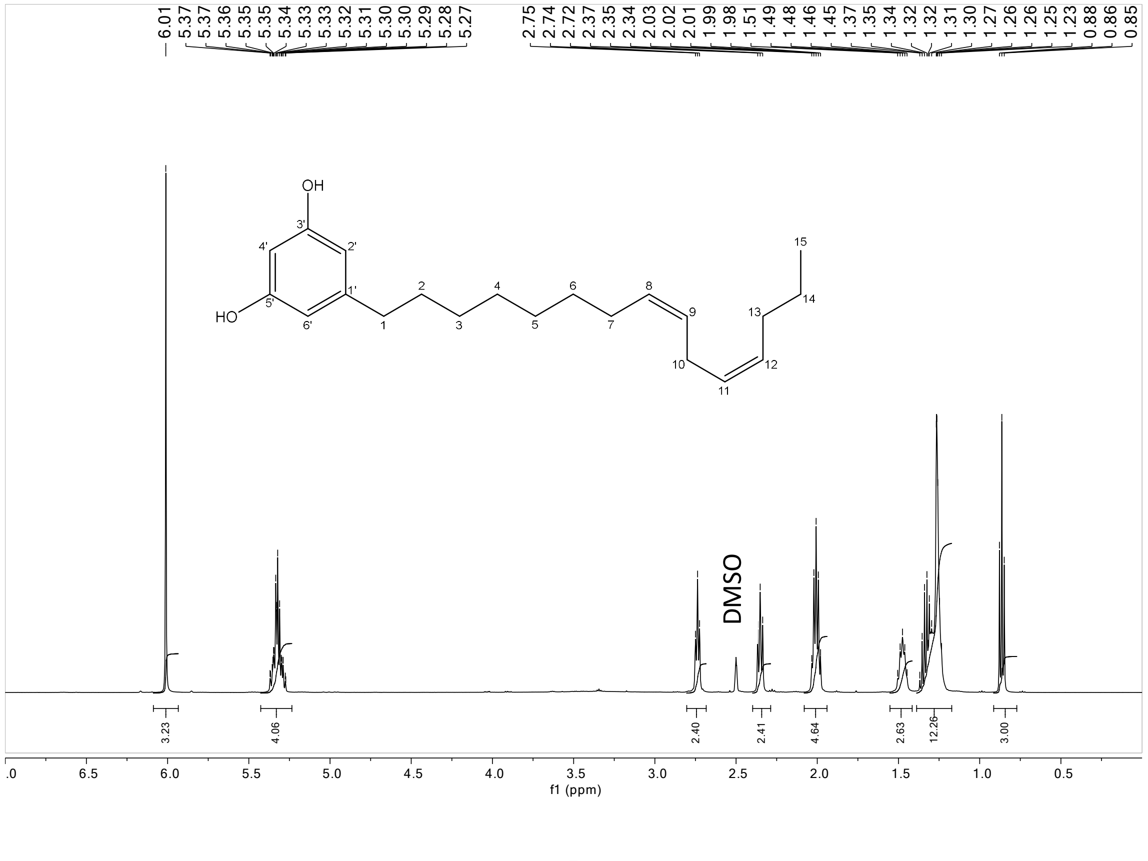

Figure 7S. ^{1}H NMR spectrum of compound **2** in DMSO-*d*6.

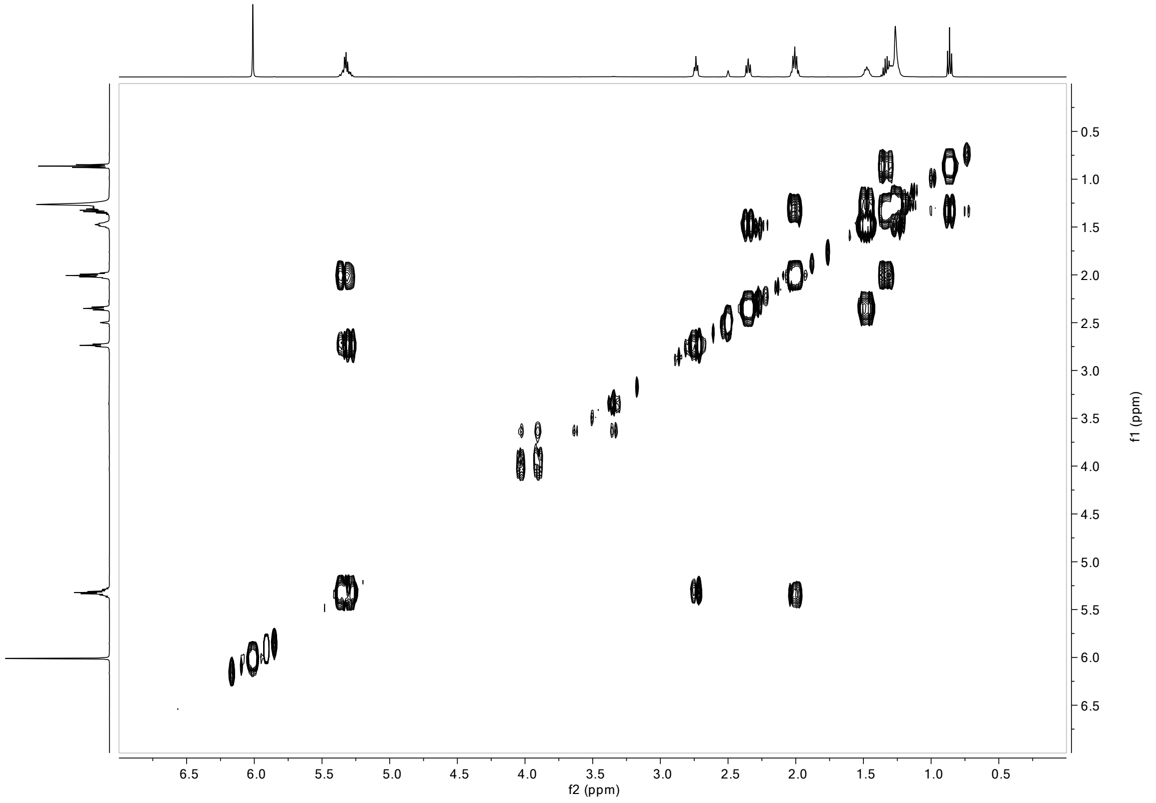

Figure 8S. COSY NMR spectrum of compound **2** in DMSO-*d*6.

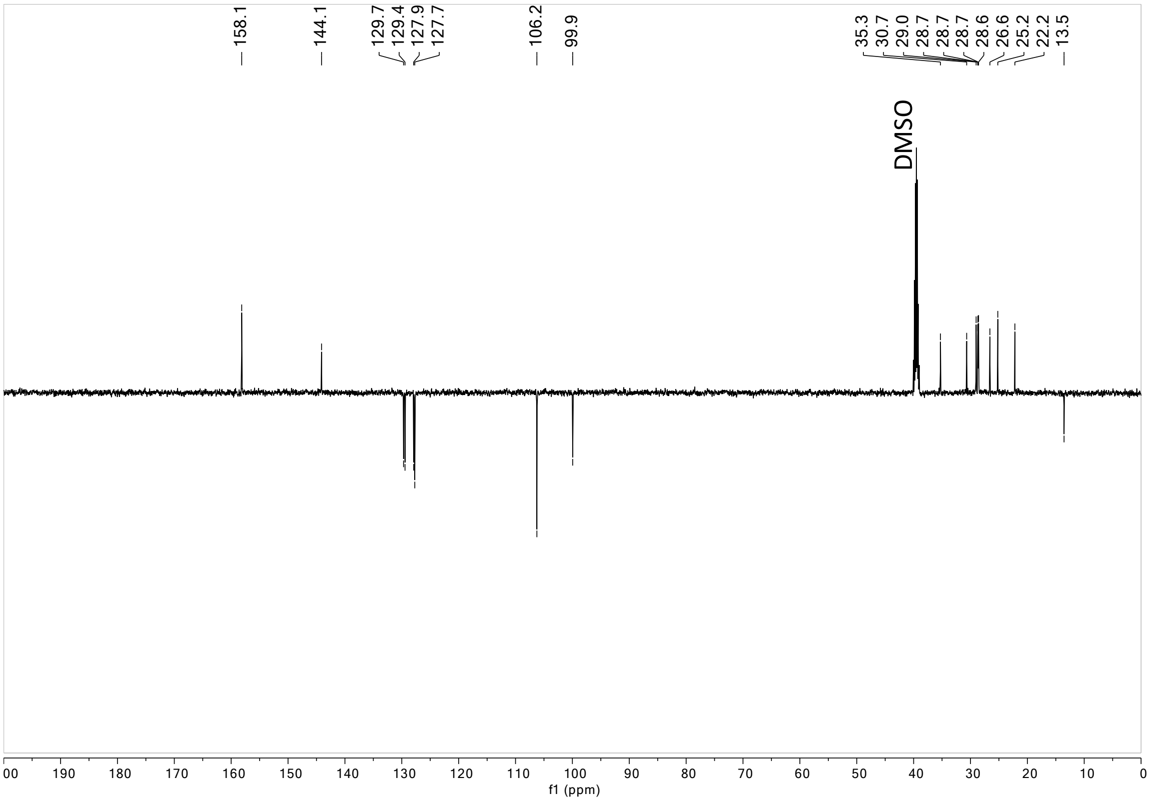

Figure 9S. ^{13}C NMR spectrum of compound **2** in DMSO-d_6.

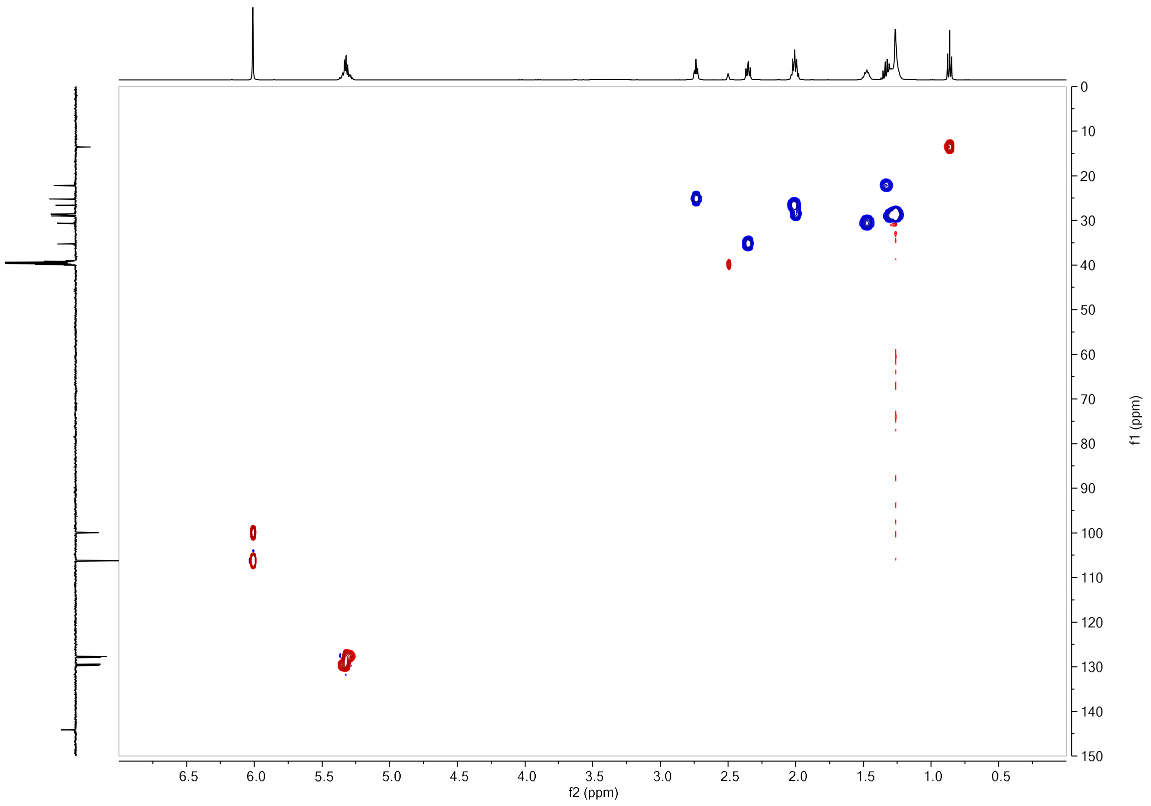

Figure 10S. Edited HSQC NMR spectrum of compound **2** in DMSO-d_6.

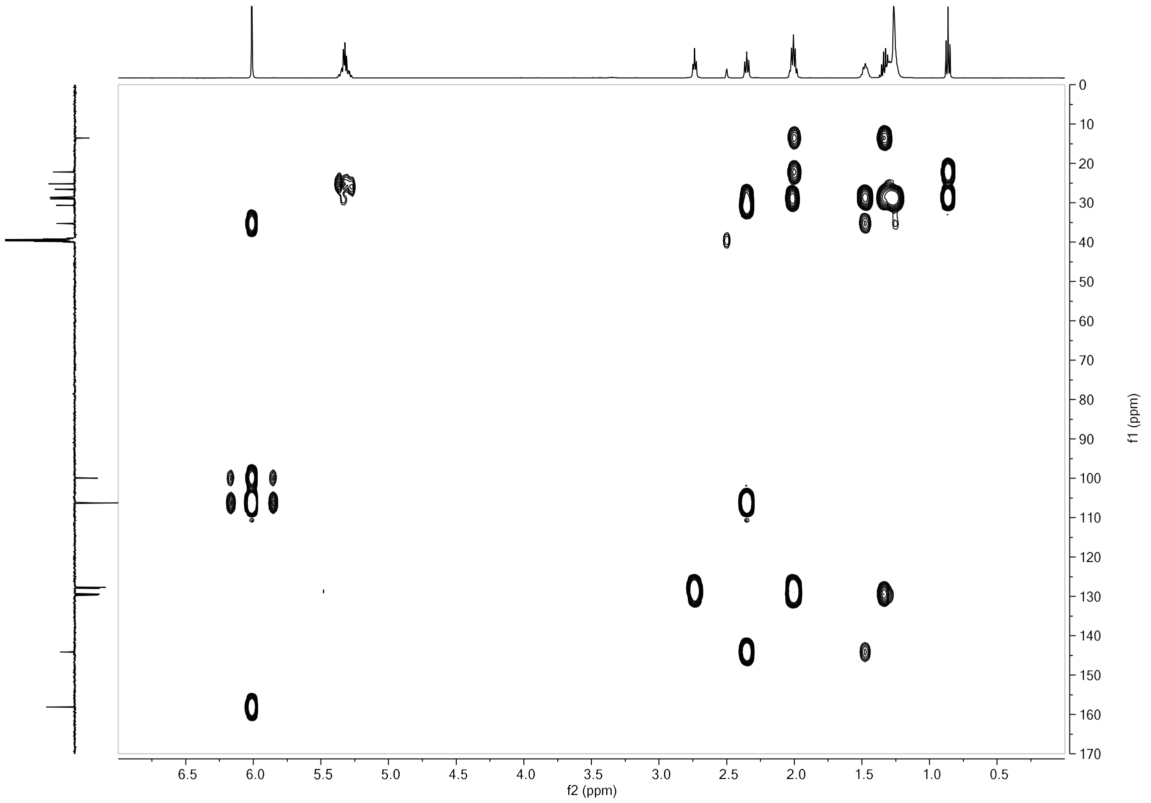

Figure 11S. HMBC NMR spectrum compound **2** in DMSO-*d*6.

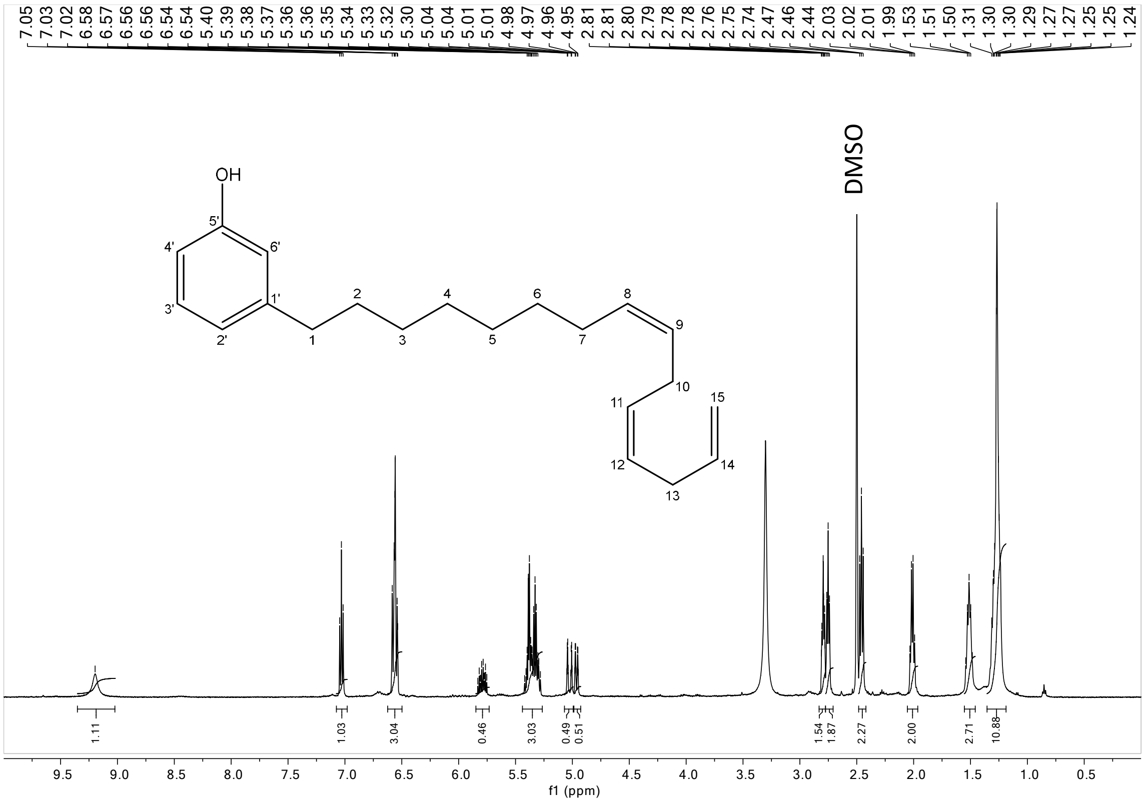

Figure 12S. ^{1}H NMR spectrum of compound **3** in DMSO-*d*6.

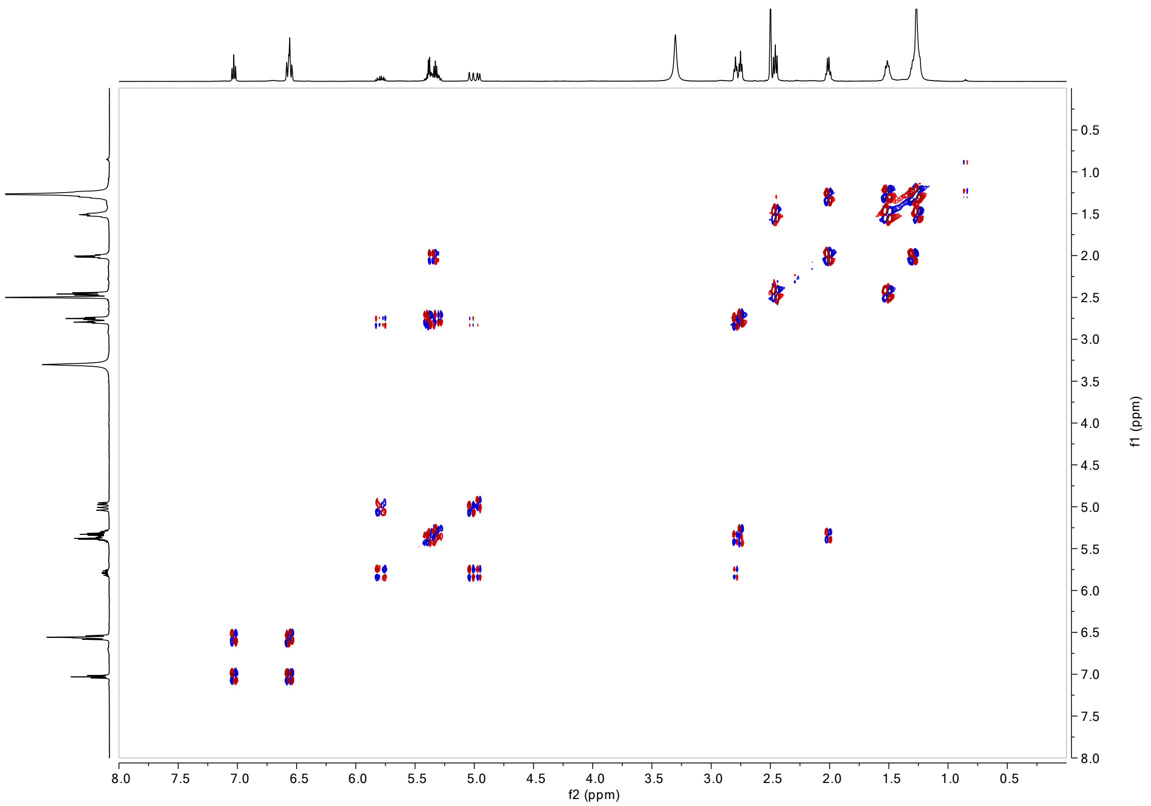

Figure 13S. DQF-COSY NMR spectrum of compound **3** in DMSO-*d*$_6$.

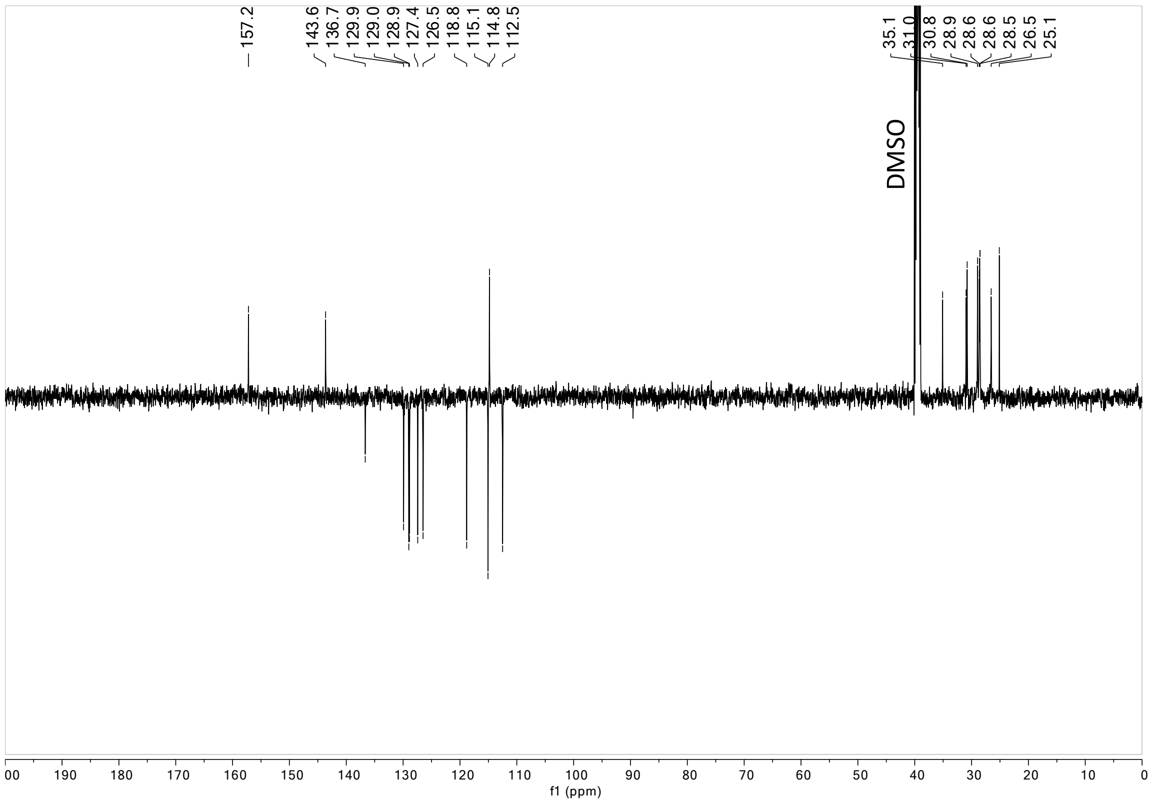

Figure 14S. ^{13}C NMR spectrum of compound **3** in DMSO-*d*$_6$.

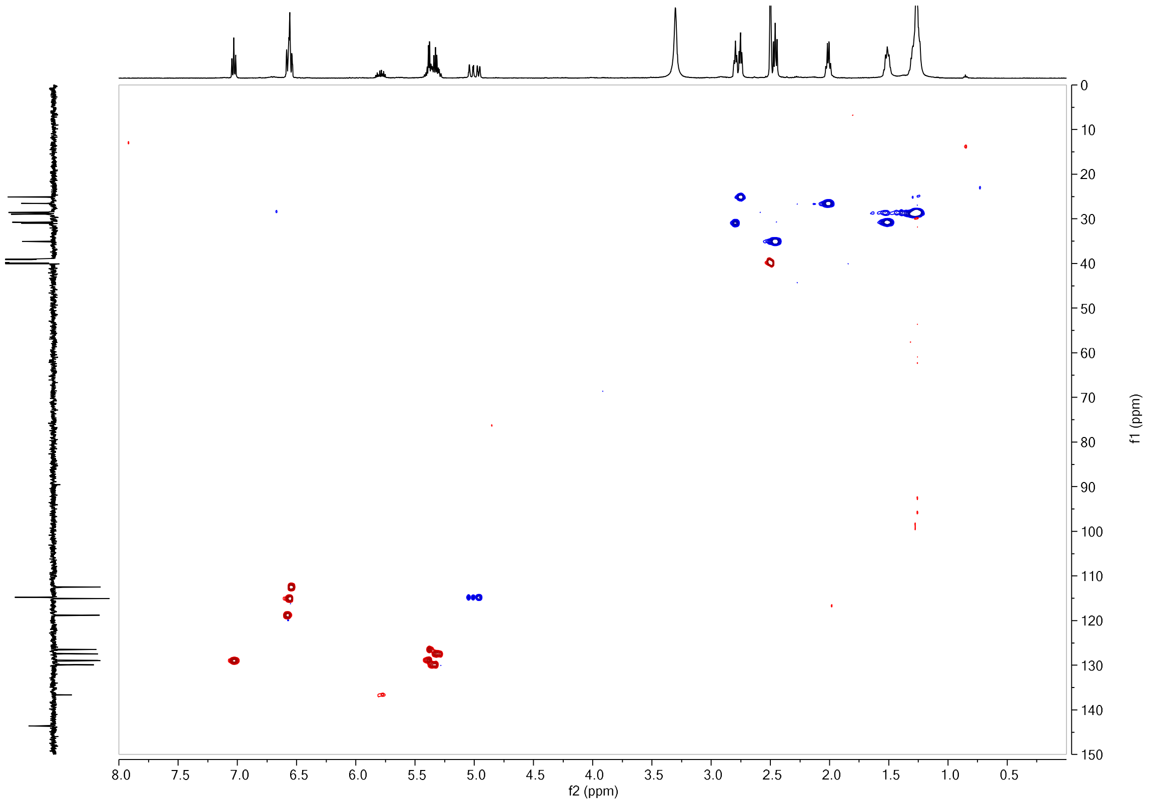

Figure 15S. Edited HSQC NMR spectrum of compound **3** in DMSO-*d*6.

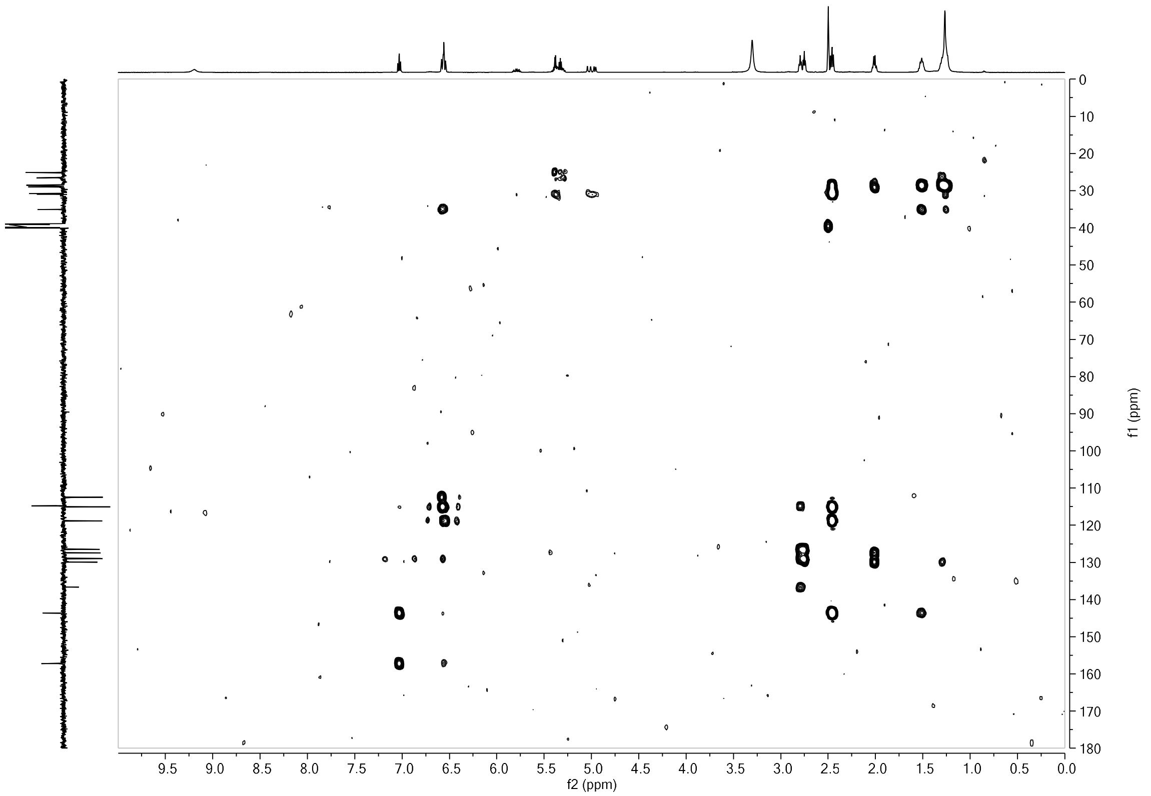

Figure 16S. HMBC NMR spectrum of compound **3** in DMSO-*d*6.

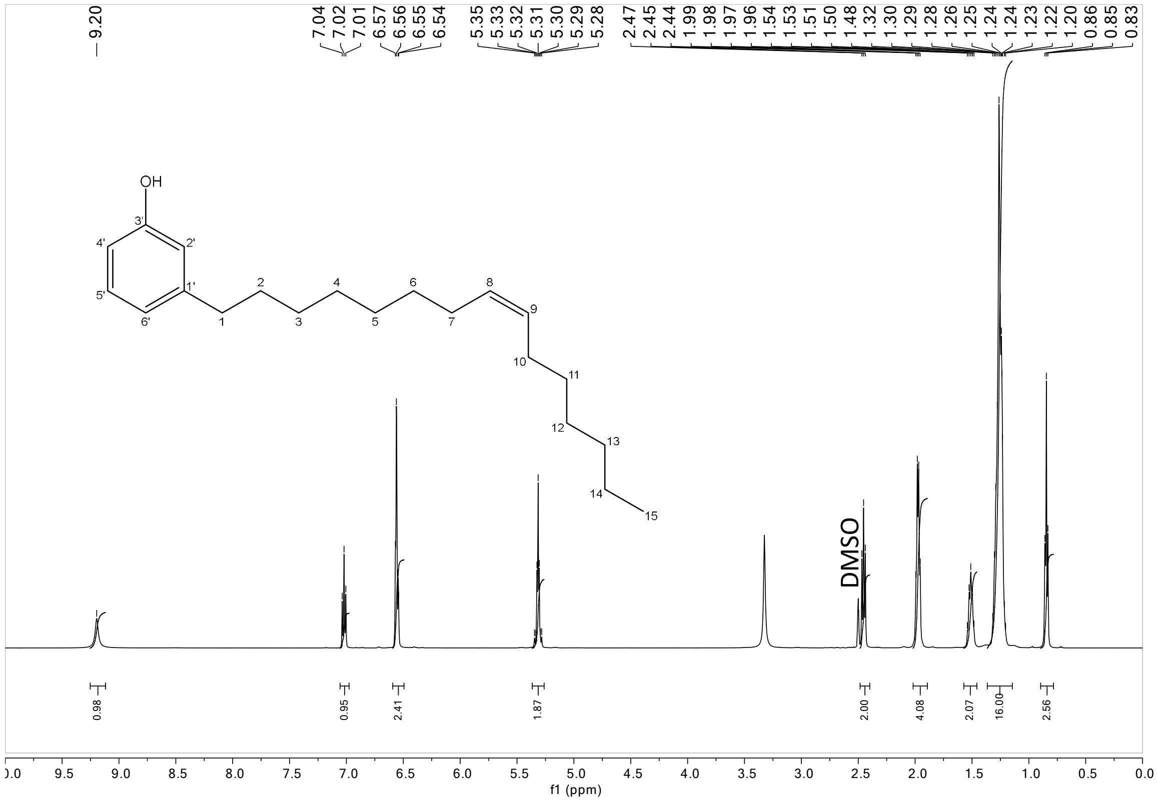

Figure 17S. ^{1}H NMR spectrum compound **4** in DMSO-*d*6.

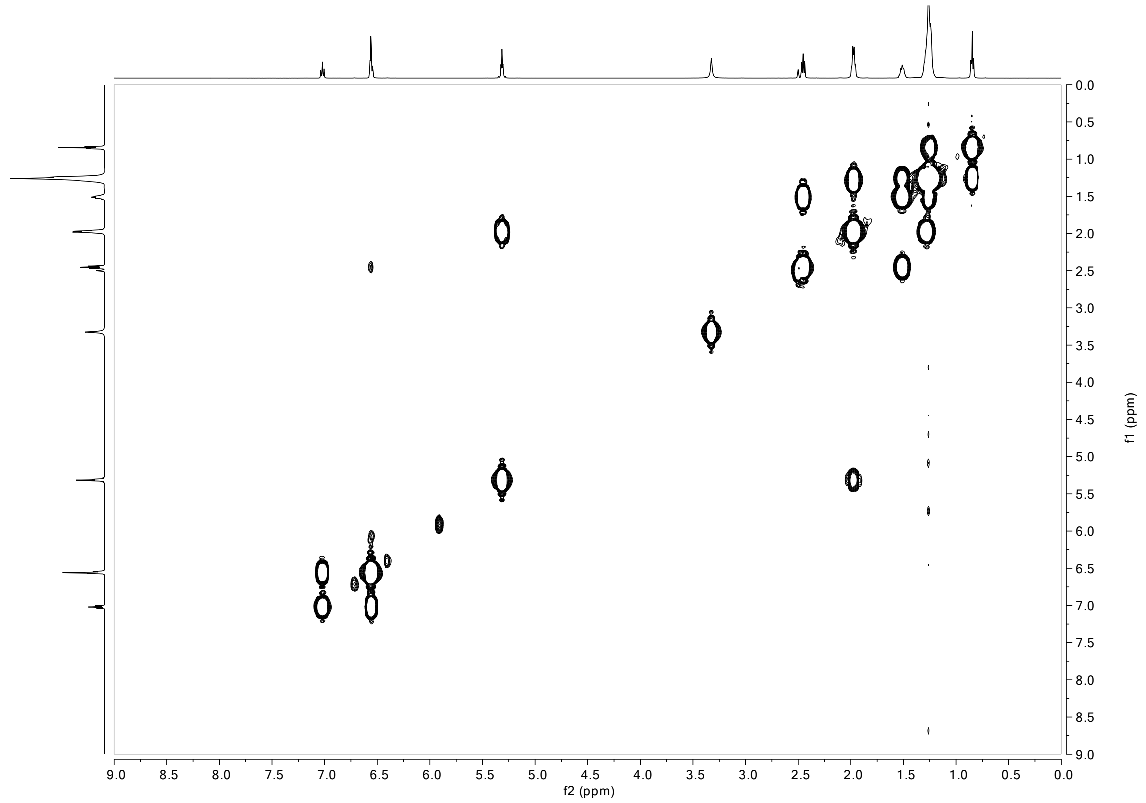

Figure 18S. COSY NMR spectrum of compound **4** in DMSO-*d*6.

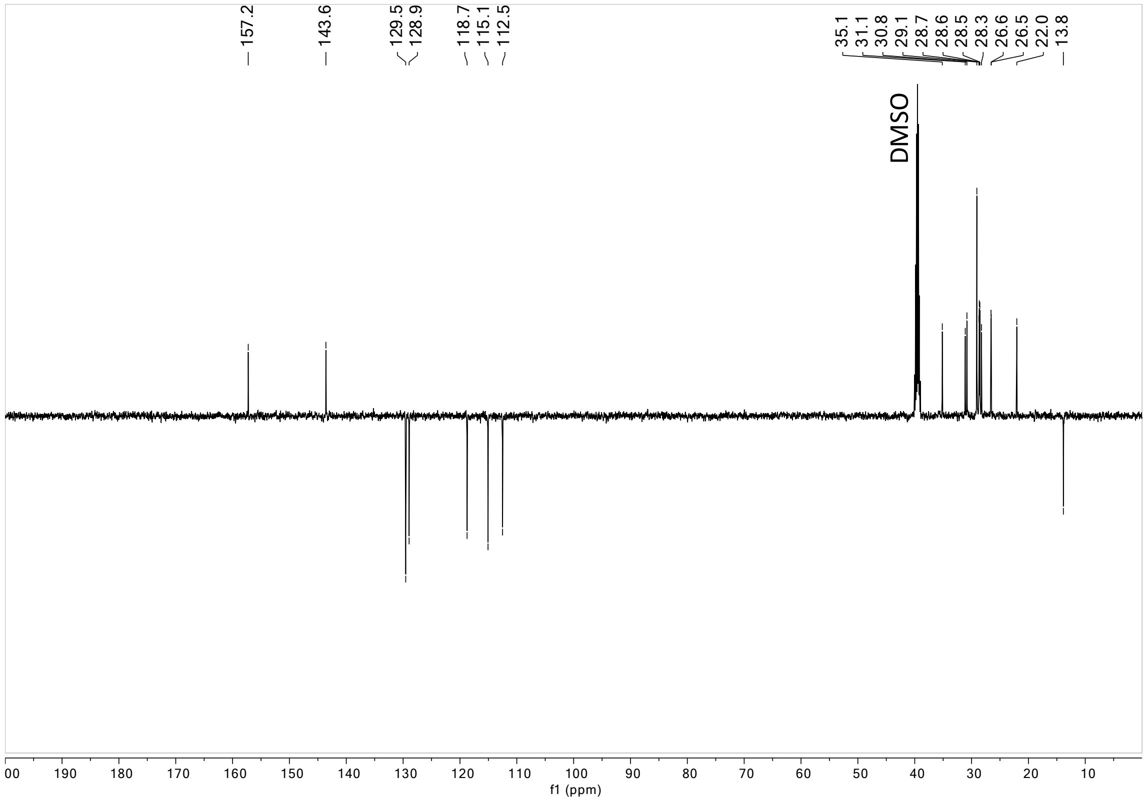

Figure 19S. ^{13}C NMR spectrum of compound **4** in DMSO-*d*$_6$.

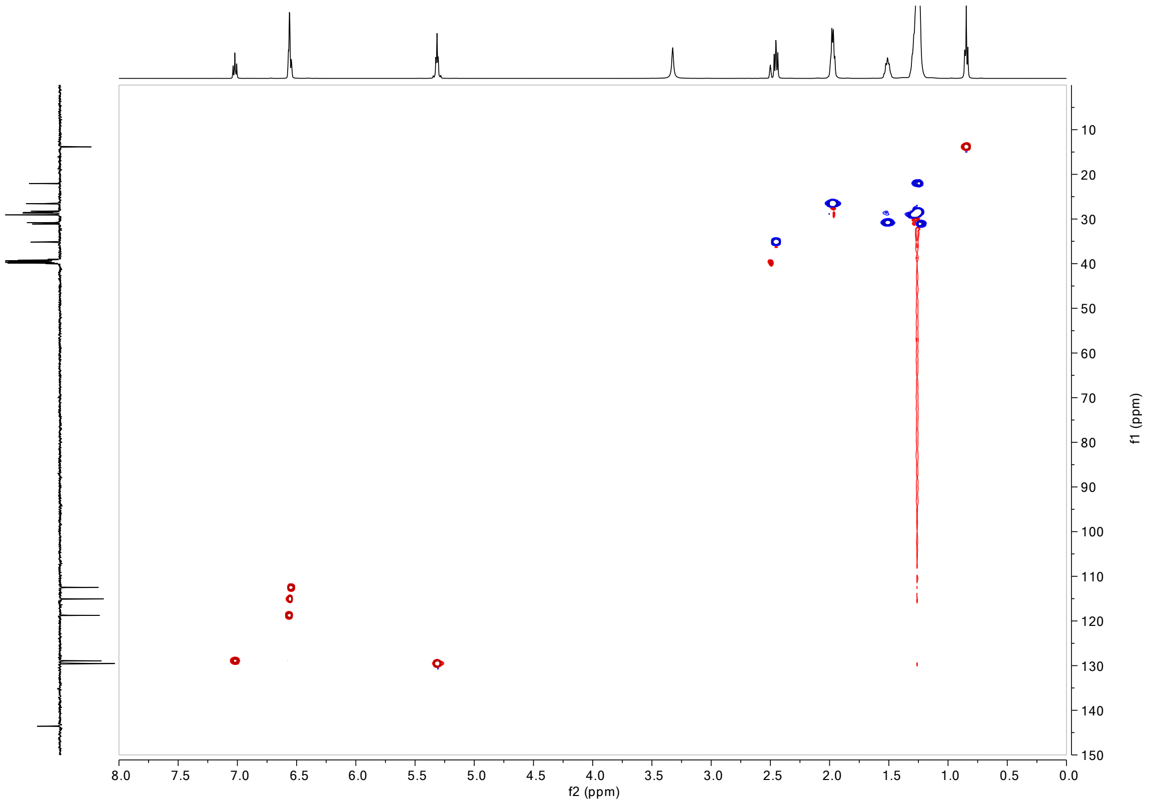

Figure 20S. Edited-HSQC NMR spectrum of compound **4** in DMSO-*d*$_6$.

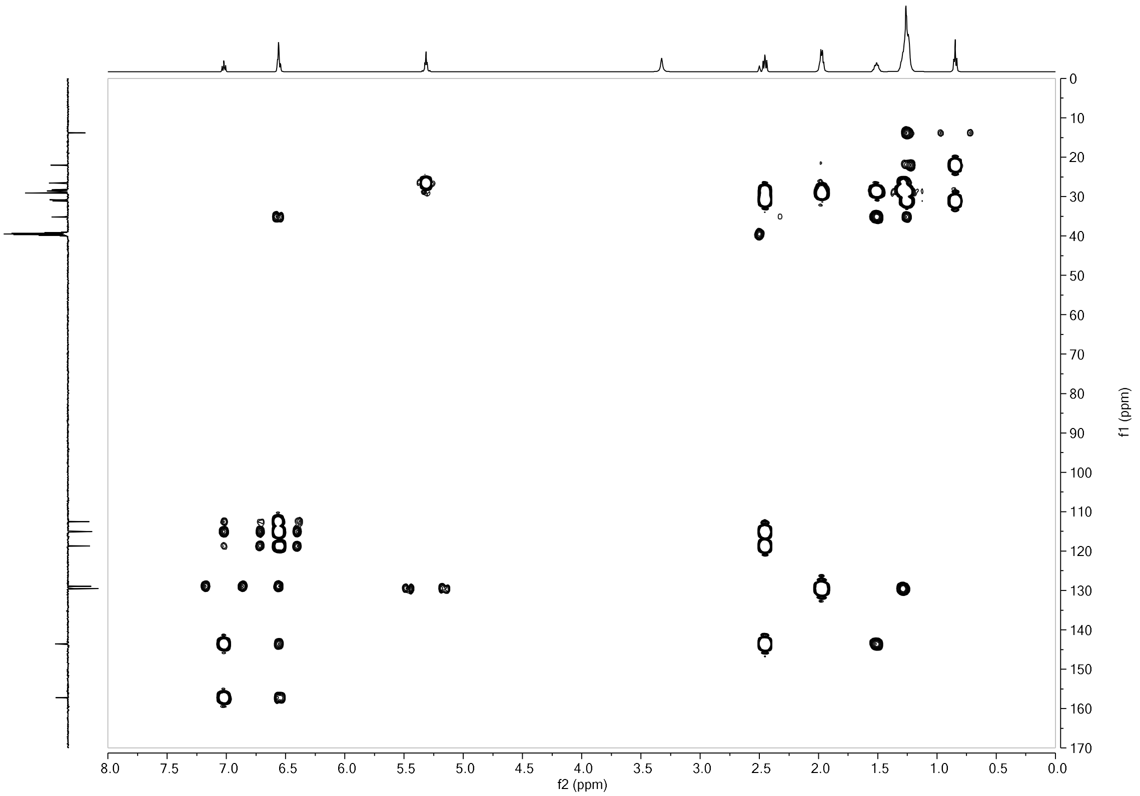

Figure 21S. HMBC NMR spectrum of compound **4** in DMSO-d_6.

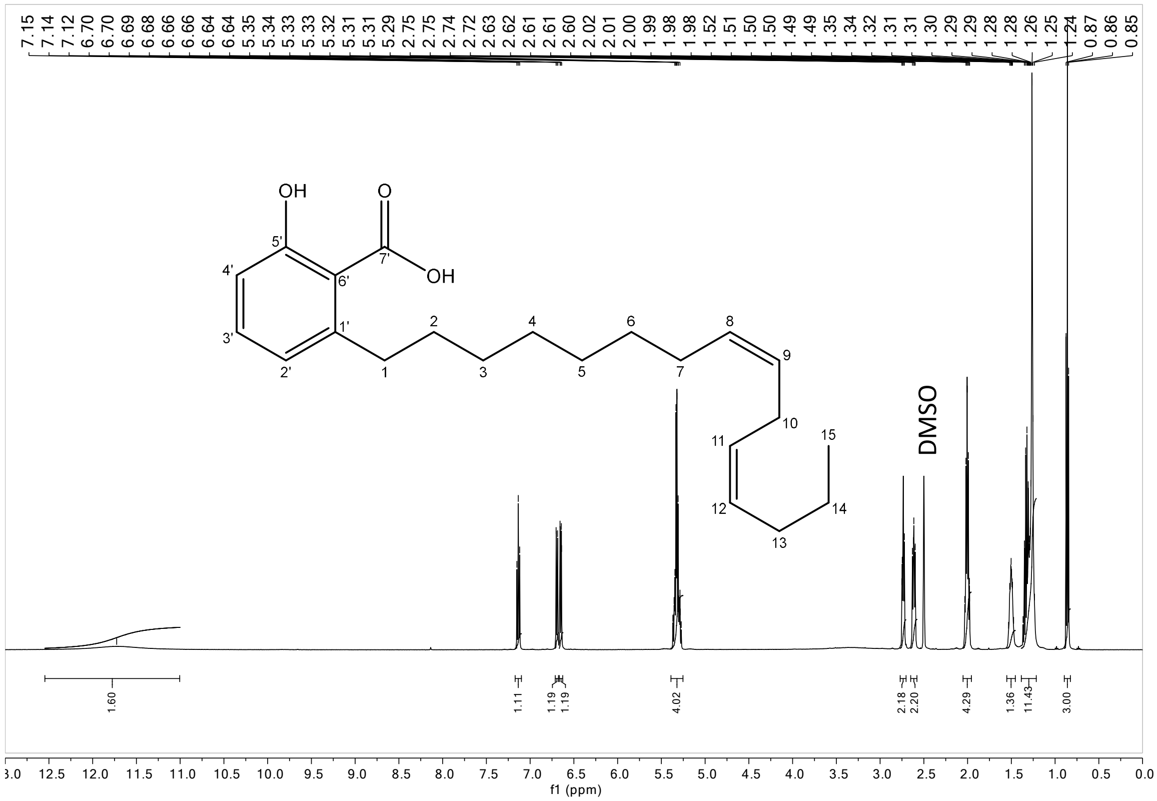

Figure 22S. ^{1}H NMR spectrum of compound **5** in DMSO-d_6.

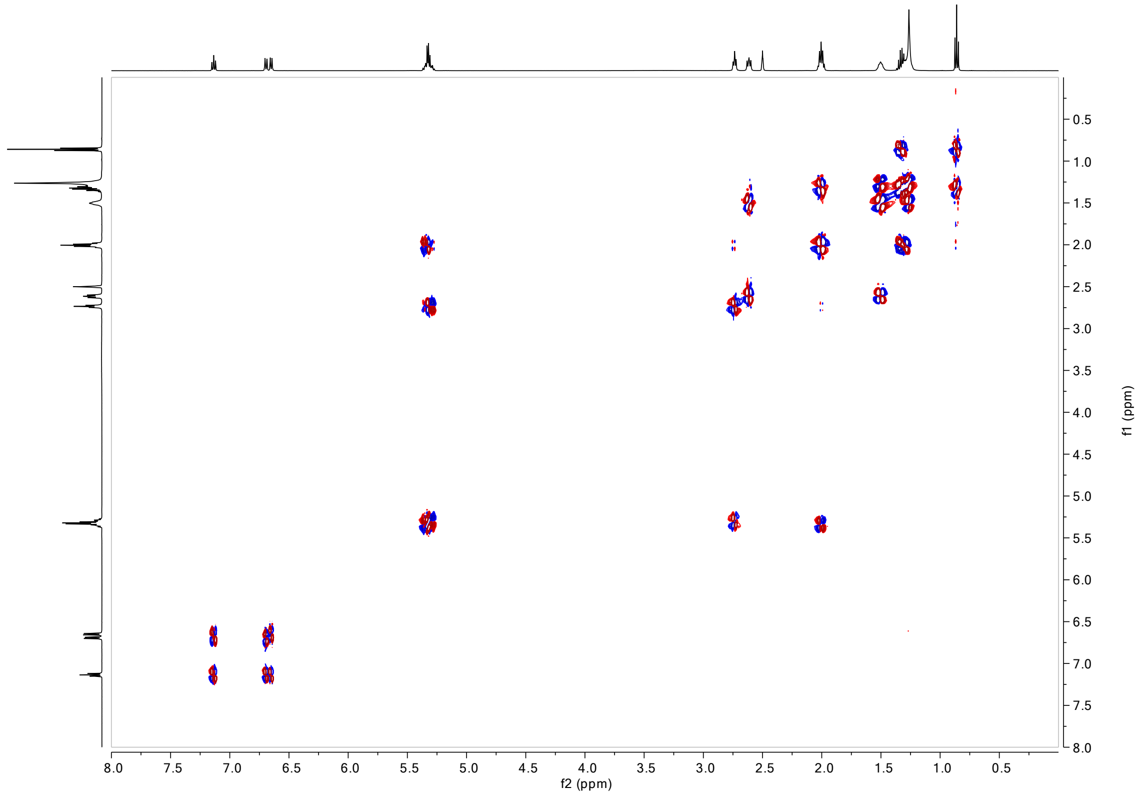

Figure 23S. DQF COSY NMR spectrum of compound **5** in DMSO-d_6.

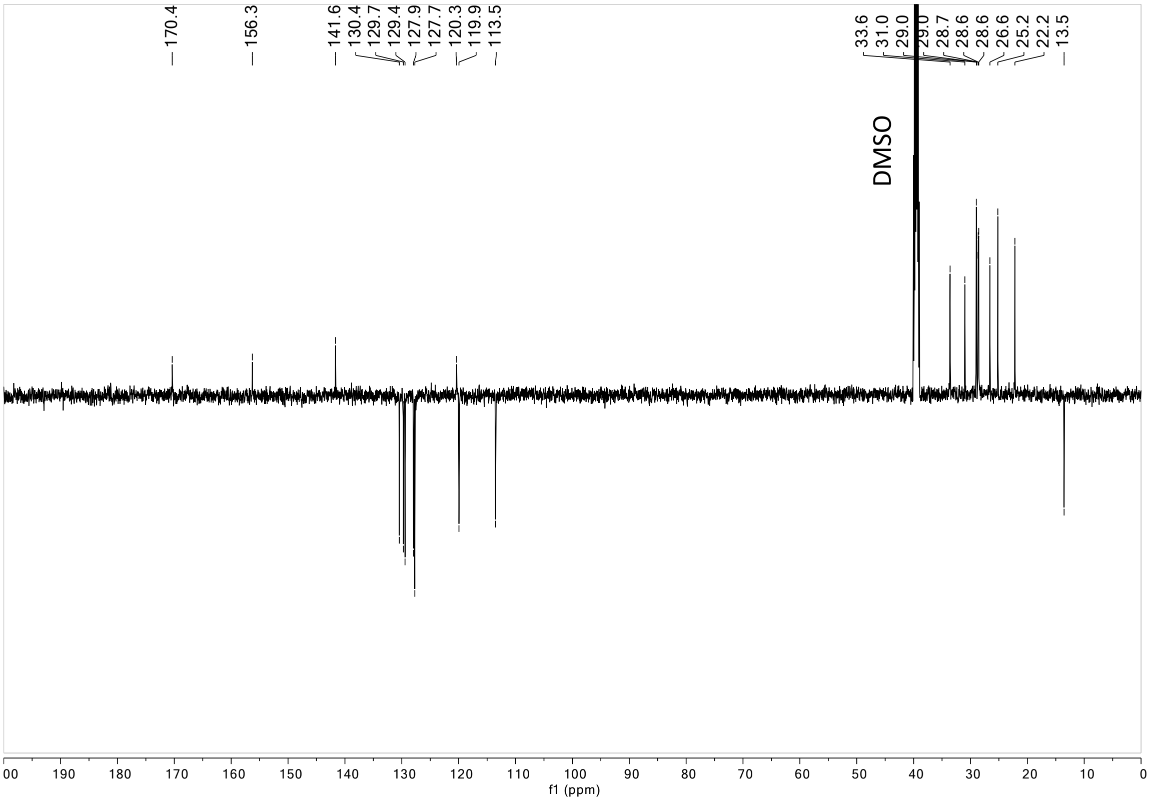

Figure 24S. ^{13}C NMR spectrum of compound **5** in DMSO-d_6.

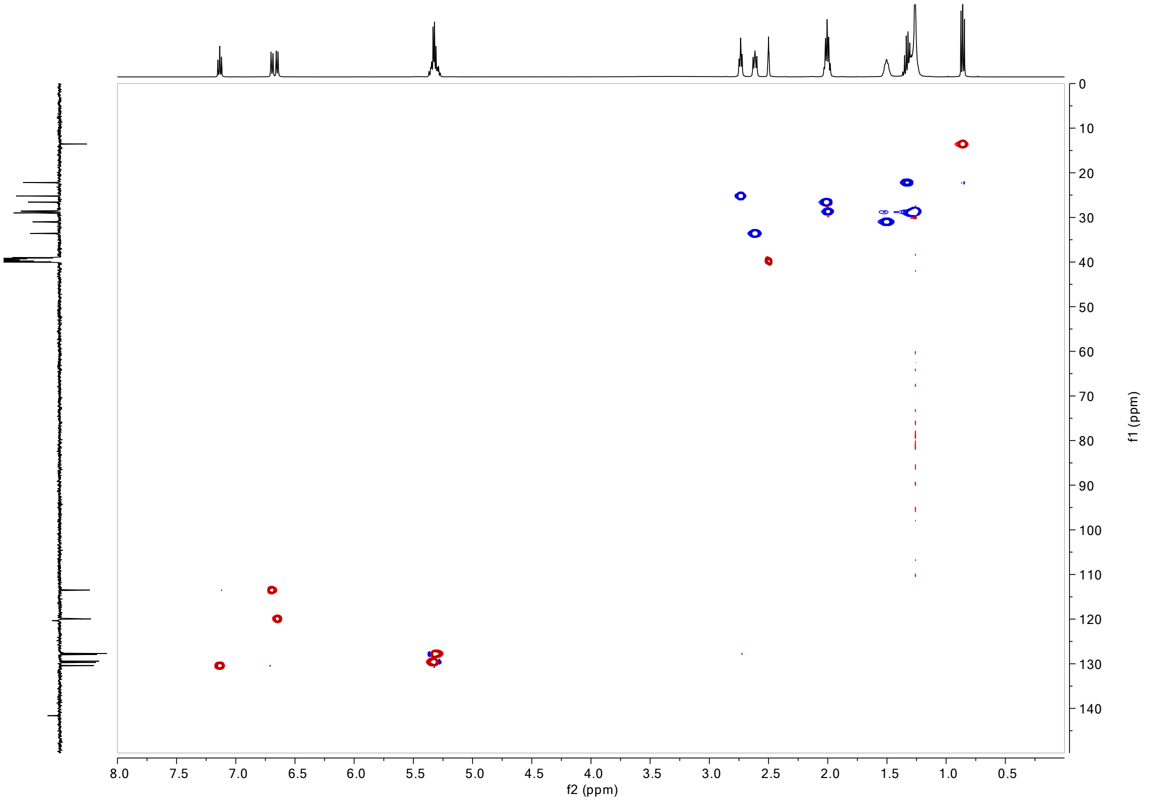

Figure 25S. Edited-HSQC NMR spectrum of compound **5** in DMSO-d_6.

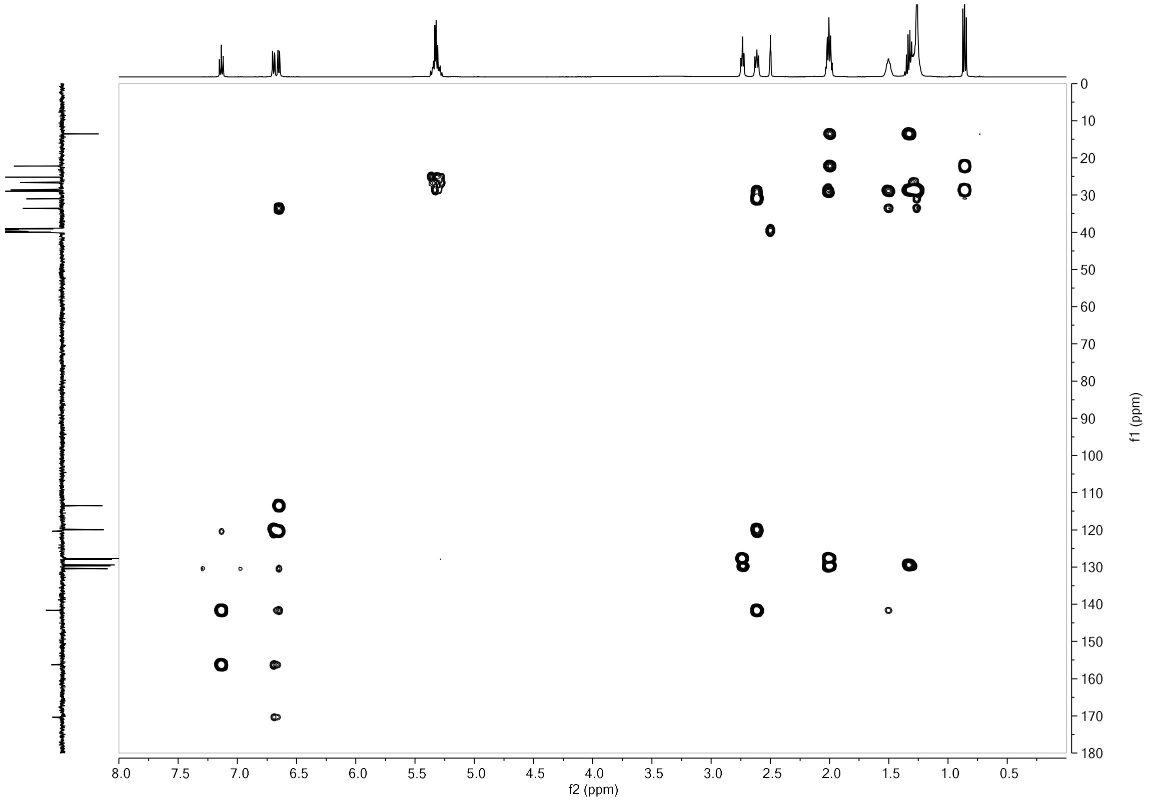

Figure 26S. HMBC NMR spectrum of compound **5** in DMSO-d_6.

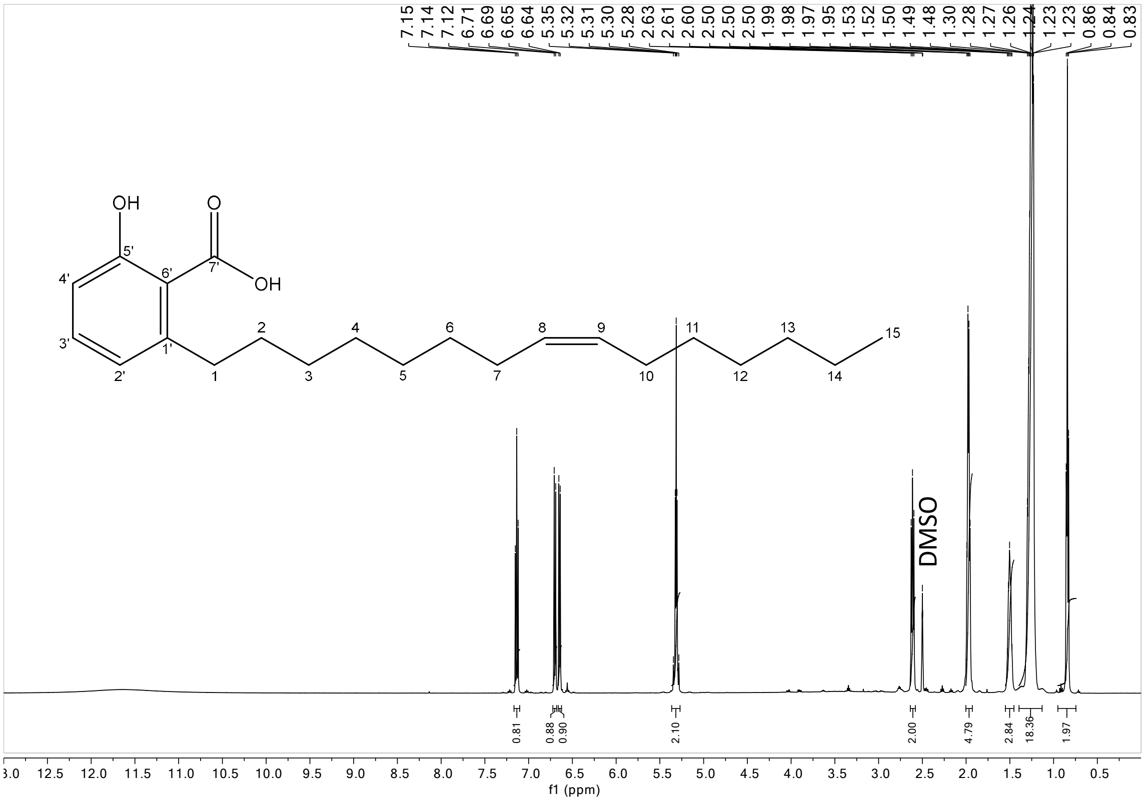

Figure 27S. ^{1}H NMR spectrum of compound **6** in DMSO-*d*6.

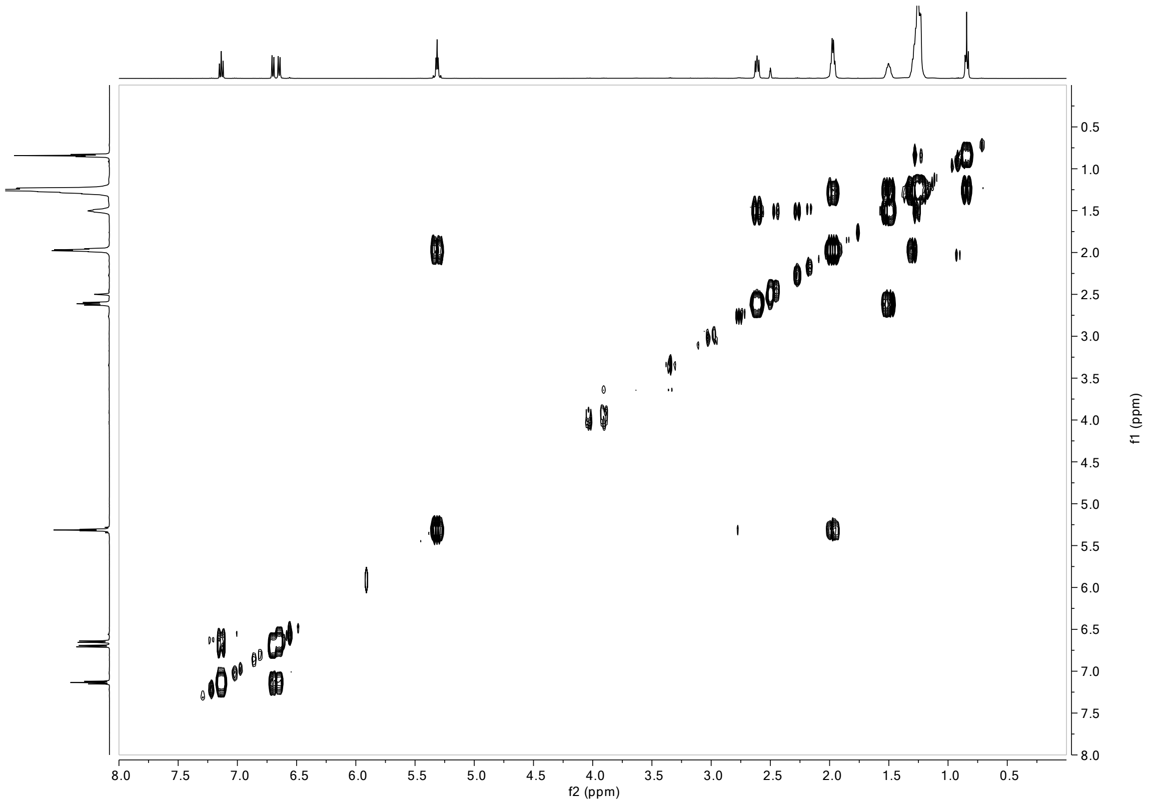

Figure 28S. COSY NMR spectrum of compound **6** in DMSO-*d*6.

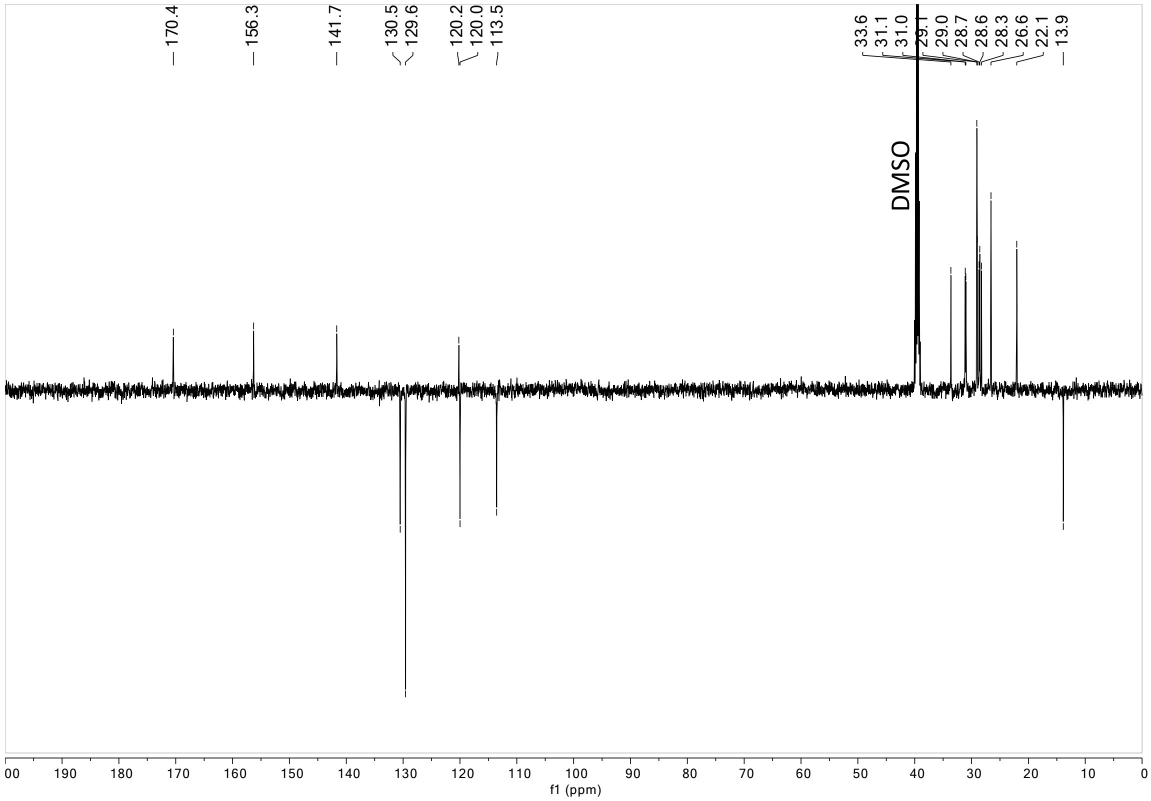

Figure 29S. ^{13}C NMR spectrum of compound **6** in DMSO-*d*$_6$.

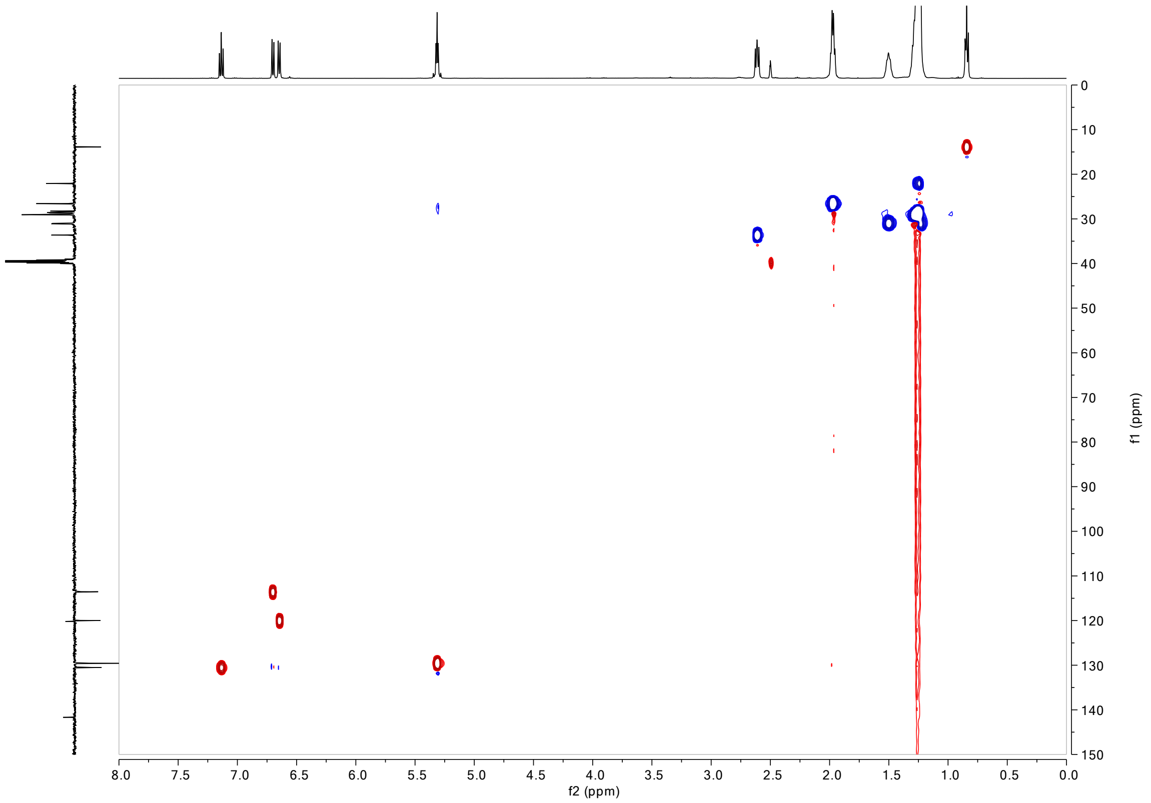

Figure 30S. Edited-HSQC NMR spectrum of compound **6** in DMSO-*d*$_6$.

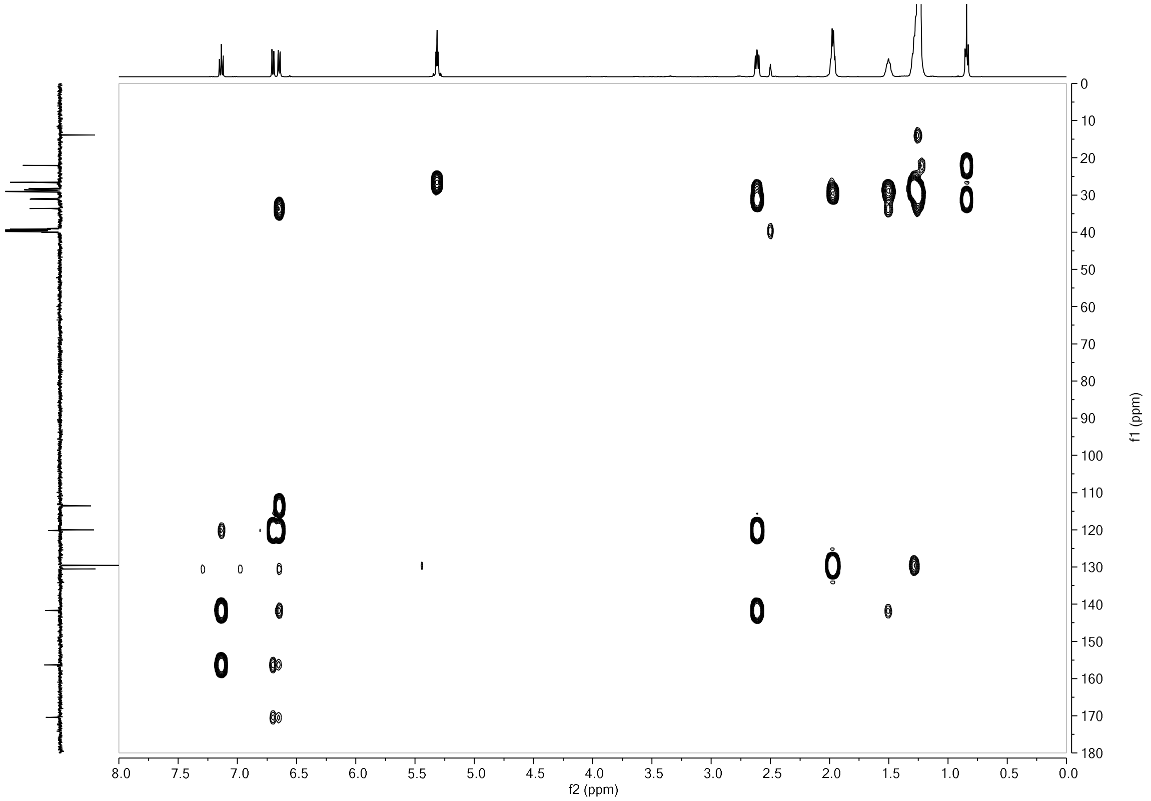

Figure 31S. HMBC NMR spectrum of compound **6** in DMSO-d_6.
